# An efficient machine learning approach for predicting concrete chloride resistance using a comprehensive dataset

**DOI:** 10.1038/s41598-023-42270-3

**Published:** 2023-09-12

**Authors:** Maedeh Hosseinzadeh, Seyed Sina Mousavi, Alireza Hosseinzadeh, Mehdi Dehestani

**Affiliations:** Faculty of Civil Engineering, Babol Noshirvani University of Technology, 484, Babol, 47148-71167 Iran

**Keywords:** Engineering, Civil engineering, Composites

## Abstract

By conducting an analysis of chloride migration in concrete, it is possible to enhance the durability of concrete structures and mitigate the risk of corrosion. In addition, the utilization of machine learning techniques that can effectively forecast the chloride migration coefficient of concrete shows potential as a financially viable and less complex substitute for labour-intensive experimental evaluations. The existing models for predicting chloride resistance encounter two primary challenges: the constraints imposed by a limited dataset and the absence of certain input variables. These factors collectively contribute to a decrease in the overall effectiveness of these models. Therefore, this study aims to propose an advanced approach for dataset cleaning, utilizing a comprehensive experimental dataset comprising 1073 pre-existing experimental outcomes. The proposed model for predicting the chloride diffusion coefficient incorporates various input variables, such as water content, cement content, slag content, fly ash content, silica fume content, fine aggregate content, coarse aggregate content, superplasticizer content, fresh density, compressive strength, age of compressive strength test, and age of migration test. The utilization of the artificial neural network (ANN) technique is also employed for the processing of missing data. The current supervised learning incorporates both regression and classification tasks. The efficacy of the proposed models for accurately predicting the chloride diffusion coefficient has been effectively validated. The findings indicate that the XGBoost and SVM algorithms exhibit superior performance compared to other regression prediction algorithms, as evidenced by their high R2 scores of 0.94 and 0.91, respectively. In relation to classification algorithms, the findings demonstrate that the Random Forest, LightGBM, and XGBoost models exhibit the highest levels of accuracy, specifically 0.93, 0.96, and 0.97, respectively. Furthermore, a website has been developed that is capable of predicting the chloride migration coefficient and chloride penetration resistance of concrete.

## Introduction

The concrete composition considerably impacts concrete's mechanical and durability characteristics^1,2^. Despite the mechanical properties, understanding the effect of concrete composition on durability is still challenging for researchers in this field^3^. The emergence of a new generation of concretes (including recycled, innovative, and sustainable materials) has increased this concern about the durability of concrete structures. The durability of reinforced concrete (RC) structures is considerably influenced by chloride penetration, especially in coastal and chloride-rich environments, resulting in a severe corrosion issue for steel reinforcement or steel fibers^4^. This corrosion phenomenon causes considerable economic loss and environmental influence in the construction industry. Various resources cause chloride attacks, such as de-icing salt, seawater, and groundwater. Different parameters substantially affect the chloride resistance of RC members, including concrete composition, temperature, relative humidity, and carbonation. Among these variables, concrete composition plays a major role in controlling internal damage of chloride attack. Previous studies strongly emphasized that concrete composition directly affects the fresh, mechanical, and durability characteristics^2,5^. Concrete composition means the water-to-binder ratio (W/B), water content, cement content, cement type, aggregate content, aggregate type, fillers, nanomaterials, mineral additives, and chemical admixtures. Each of these ingredients affects the concrete porosity. Achieving a reliable concrete mixture with the lowest permeability to prevent the penetration of the chloride ions is one of the main practical solutions to mitigate internal chloride attack damages. For instance, different supplementary cementitious materials (SCMs), fillers, and nanomaterials can be used to obtain an efficient concrete mixture resistant to chloride attack. However, as innovative and new concrete generations have been introduced in recent years, standards recommended experimental rapid tests to determine the performance of these new cementitious composites against chloride attack, including salt ponding, bulk diffusion, and rapid chloride permeability tests. These tests are time-consuming (more than 28 days) and require an adequate material, researchers, and testing equipment budget. Hence, a practical solution and an extensive experimental program are necessary to determine the efficiency of a proposed mixture for concrete members exposed to chloride attack.

Experimental tests considered different parameters to determine the chloride resistance of mixtures. As mentioned in Eq. (1), Fick’s second law and its analytical solution have usually been used to explain the chloride diffusion of concrete for measuring the service life design of RC members^6,7^, where $C(x. t)$ is the chloride ions concentration, $t$ and $x$ denote the time and position from the exposed concrete surface, respectively, ${D}_{C}$ is the chloride diffusion coefficient, C_0_ is the initial chloride concentration in concrete, and C_s_ is the apparent surface chloride concentration.

1 $$\frac{{\partial C\left( {x. t} \right)}}{\partial t} = D_{C} \frac{{\partial^{2} C\left( {x. t} \right)}}{{\partial x^{2} }}$$

2 $$C\left( {x. t} \right) = C_{0} + \left( {C_{S} - C_{0} } \right)\left[ {1 - erf\left( {\frac{x}{{2\sqrt {D_{C} t} }}} \right)} \right]$$

Among these empirical parameters, the chloride diffusion coefficient plays a critical role in showing the chloride resistance of concrete mixtures. Different parameters affect ${D}_{C}$ as a time-dependent material characteristic, including concrete composition, curing conditions, chloride exposure time, and exposure location^8^. Many experimental test methods were used to determine the chloride diffusion coefficient. As recommended by ASTM C1556–11^9^ and NT Build 443^10^, bulk diffusion tests are time-consuming tests where concrete samples are exposed to a chloride solution for a long period. In this context, Nordic standard NT Build 492^11^ proposed an accelerated test method, where chloride ions infuse the concrete at high rates caused by the applied electric field. The chloride diffusion coefficient measured by this method is denoted as the “non-steady-state migration coefficient (${D}_{nssm}$)”. Standard criteria for classification of chloride penetration resistance of concrete based on this NT Build 492^11^ method compared to ASTM C1202^12^ is mentioned in Table 1. ASTM C1202^12^ considers five categories for chloride ion penetrability based on the passed electric charge (PEC), while ${D}_{nssm}$ was used in NT Build 492^11^. Although the literature severally confirmed the accuracy of this method, the test procedure needs a practiced operator and accurate resources and facilities. Although this method's experimental results are precise, conducting this test for each concrete mixture for RC projects is not feasible and practical due to the long-term immersion period and costs. Hence, an efficient and practical solution is needed to determine the chloride resistance of concrete mixture for each project. Accordingly, it is crucial to advance a feasible and accurate predicting model that specify the chloride diffusion coefficients using concrete composition.

**Table 1:** Standard criteria for the classification of chloride penetration resistance of concrete.

Standard				
NT Build 492^13^			ASTM C1202^12^	
Chloride migration coefficient (*D*_*nssm*_) ($\times {10}^{-12} {m}^{2}/s$)	Chloride penetration resistance	Data encoding	Charge passed (Coulombs)	Chloride ion penetrability
$>$ 15	Low	0	$>$ 4000	High
10–15	Moderate	1	2000–4000	Moderate
5–10	High	2	1000–2000	Low
2.5–5	Very high	3	100–1000	Very low
$<$ 2.5	Extremely high	4	$<$ 100	Negligible

Recently, in the last few years, the use of artificial intelligence (AI) to predict cementitious composites' mechanical and durability characteristics has gained significant speed. However, specific types of concrete were considered for each study, whereas a comprehensive dataset was required in a unified AI model. Additionally, the number of datasets is another critical issue of these investigations. For instance, Amin et al. (2023)^14^ presented an effective predicting method for the strength of sustainable concrete utilizing rice husk ash (RHA) as supplementary cementitious materials using machine learning (ML) algorithms. Hosseinzadeh et al. (2023)^15^ used ML techniques (both regression and classification algorithms), to forecast the mechanical properties of fly ash-based recycled aggregate concrete (FARAC). Jiao et al. (2023)^16^ employed ML to predict the compressive strength of concrete with carbon nanotubes as nanomaterials. In this context, Zheng et al. (2023)^17^ developed an ML model to predict the compressive strength of silica fume concrete. Ullah et al. (2022)^18,19^ used the ML method to predict dry density and compressive strength of lightweight foamed concrete. In this field, Khan et al. (2021)^20^ used the ML method to formulate the depth of wear of fly-ash concrete. Song et al. (2021)^21^ worked on presenting an ML model to predict the compressive strength of concrete with fly ash admixture. Farooq et al. (2021)^22^ used the ML method to formulate the strength of self-compacting concrete (SCC). Ahmad et al. (2021)^23^ employed the ML method to forecast the compressive strength of concrete at high temperatures considering 207 experimental datasets. Ahmad et al. (2021)^24^ presented an ML model along with gene expression programming (GEP) and artificial neural network (ANN) to predict the compressive strength of recycled coarse aggregate (RCA)-based concrete. Farooq et al. (2021)^25^ employed ML algorithms with individual learners and ensemble learners (bagging, boosting) to forecast the mechanical properties of high-performance concrete using waste materials. Ahmad et al. (2021)^26^ used ML to predict the compressive strength of fly ash-based concrete. In this field, Khan et al. (2021)^27^ used soft-computing techniques, including random forest regression and gene expression programming to present a prediction model for the compressive strength of fly ash-based geopolymer concrete. Other studies in the literature also used AI methods in predicting the mechanical characteristics of cementitious composites^28,29^.

Accordingly, there has also been a growing interest in using AI techniques for predicting chloride diffusion coefficients, such as ANN, fuzzy inference systems, and ML methods. Table 2 comprehensively gathered all available AI models introduced by researchers for chloride diffusion coefficient. Thirty-four predicting models were proposed by the literature, considering different input variables, output factors, dataset numbers, and concrete types. Water-to-binder ratio (W/B), water content, cement content, fly ash (FA) content, silica fume (SF) content, ground granulated blast-furnace slag (GGBFS) content, superplasticizer dosage (SP), fine aggregate content (sand), coarse aggregate content, submerged duration time, curing age, air-entraining admixture, viscosity modifying admixtures dosage (VMA), testing age, concrete compressive strength (${f}_{c}$), specific surfaces of FA, environmental conditions, diffusion rate, etc., are the input variables considered in the current predicting models reported by the literature. Also, different output variables were considered in these studies, such as non-steady-state migration coefficient and surface chloride concentration (Table 2). Along with obtaining a predicting model for the chloride resistance of concrete, previous AI studies reported some critical and conflicting findings based on their soft computing models. Generally, two different types of AL models were presented by the literature, including (a) a model for only a specific type of concrete mixture; and (b) an integrated model with no limitation for concrete type. Although most of the models developed by previous studies concentrate on the first group, the literature recently introduced some valuable models for the second category. Regarding the first model type, Parichatprecha & Nimityongskul (2009)^30^ used the ANN method to predict chloride ions permeability using 86 datasets and 8 input variables for high-performance concrete (HPC) mixtures with compressive strength ranging from 30 to 120 MPa. They reported that the optimum cement content for chloride-resistant HPC is 450–500 kg/m^3^. Also, based on their model, the chloride resistance of HPC can be significantly improved using both SF and FA (around more than 20% cement replacement). Song & Kwon (2009)^31^ slightly increased the dataset (120 number) for HPC specimens and found that they added GGBFS along with other SCMs to the input variables to predict the diffusion coefficient of chloride ion using the ANN method. They also used the parameter “duration time in submerged condition” instead of “superplasticizer” for the input parameters. As the precision of the soft computing methods in AI is extremely dependent on the number of database, Hodhod & Ahmed (2013)^32^ used 300 datasets of HPC mixture for the ANN method. They considered 5 input variables of W/B, cement, FA, GGBFS, and curing age. Regarding self-consolidating concrete (SCC) mixtures, extensive AI models were also presented in the literature^33–35^. In this field, Ghafoori et al. (2013)^36^ used statistical and ANN models on their limited experimental database (only 24 SCC mixtures) to present a predicting model for the rapid chloride penetration test (RCPT) value choosing 6 input features of cementitious materials, W/B, coarse aggregate, fine aggregates, air-entraining admixture, and high range water reducer (HRWR). Although a general trend cannot be extracted based on their limited database, they reported that three independent parameters of cementitious materials content, W/B ratio, and coarse or fine aggregate are essential to predict the RCPT value of SCC. Accordingly, Mohamed et al. (2018)^37^ used 86 datasets of SCC mixture to present a predicting ANN model for chloride penetration level using comprehensive 11 input variables of W/C ratio, cement, GGBFS, FA, SF, water, superplasticizer, coarse aggregate, fine aggregate, age, and charge. Najimi et al. (2019)^38^ collected 72 datasets of SCC mixtures and used feed-forward ANN combined with an artificial bee colony algorithm to develop a chloride penetration model. They added air-entraining admixture and HRWR to their input variables. Kumar et al. (2020)^39^ gathered 360 datasets of SCC mixtures to predict chloride penetration using MARS and minimax probability machine regression (MPMR). They only considered three input variables of FA, SF, and temperature. In this context, the model presented by Yuan et al. (2022)^34^ revealed that temperature and SF considerably affect the model performance. In the field of high-strength concrete (HSC), Inthata et al. (2013)^40^ used 270 datasets of mixtures containing ground pozzolans to predict chloride penetration and chloride penetration depth. They added for the first time the actual concrete compressive strength to the input parameters in ANN models. Their model revealed that 40% of cement replacement by ground pozzolans shows the highest resistance to chloride penetration, especially for rice husk ash-contained concrete mixtures. In line with this research, Boğa et al. (2013)^41^ used 162 datasets of concrete containing GGBFS and calcium nitrite-based corrosion inhibitors to develop a predicting model for chloride ion permeability using a four-layered ANN and adaptive neuro-fuzzy inference system. They considered Cure type, curing period, GGBFS ratio, and calcium nitrite-based corrosion inhibitor ratio independent variables. Based on the dataset's limited range of concrete types, no general report can be extracted by their study. Regarding concrete containing high calcium FA, Marks et al. (2015)^42^ gathered 56 dataset and used ML method to predict the concrete resistance to chloride penetration. Their input parameters were water, cement, high calcium FA, and the specific surface of fly ash. Their model showed that to have a reliable chloride-resistance FA concrete, the water content should be controlled in the w ≤ 158 L/m^3^ range. However, they emphasize that the experimental mode dataset should be checked due to their limited dataset number. In the context of the limited dataset, Slika & Saad (2016)^43^ used Ensemble Kalman Filter (EnKF) on the limited dataset of NC to predict Chloride concentration. Regarding SF concrete, 162 datasets were collected by Asghshahr et al. (2016)^44^, and ANN, along with classification and regression tree (CART) methods, were utilized to predict the Chloride concentration. Only 4 input variables were considered in their models, including environmental condition, penetration depth, W/B ratio, and SF. Their models showed that the penetration depth is the most vital factor impacting the chloride concentration. However, they could not find the critical role of SF in their model. The literature also used AI models to predict chloride ion diffusion. For instance, Hoang et al. (2017)^45^ considered the ML model with the multi-gene Genetic programming (MGGP) and multivariate adaptive regression splines (MARS) to predict the chloride ion diffusion of cement mortars using mortar age, depth of measured point, diffusion dimension, the reinforcement presence as input variables. However, along with ML methods, valuable soft computing models have still been used using the ANN method on different types of concrete. In the field of AI techniques, genetic programming (GP) was also used by Gao et al. (2019)^46^ on 25 experimental databases of NC to predict chloride-ion diffusion. They found that the GP method was more efficient than the ANN model. However, based on their limited dataset, more studies should check their finding. Some previous studies also concentrated on predicting AI models of chloride resistance for recycled aggregate concrete (RAC). Ahmad et al. (2021)^47^ employed an ML model to predict the surface chloride concentration in concrete containing waste material, including gene expression programming (GEP), the decision tree (DT), and an ANN. In this field, Liu et al. (2022)^48^ used the ensemble ML method to predict the PEC gathering 226 dataset. Their soft computing technique found that water content is the most vital parameter controlling the chloride ion permeability of RAC. In this context, Jin et al. (2022)^49^ developed an ANN model to predict the chloride diffusion coefficient using 112 dataset. They considered 9 input variables consisting of cement, water, coarse aggregate, sand, water reducing agent, recycled aggregate replacement rate, W/C ratio, water absorption rate of coarse aggregate, and apparent density of coarse aggregate. However, they ignored two important parameters of recycled fines and mineral admixtures due to the lack of an appropriate dataset. Also, unnoticed the effect of cement type. Their model demonstrated that the natural fine aggregate content, along with recycled coarse aggregate, has the most substantial influence on the penetrability of RAC chloride ions. Regarding metakaolin-contained concrete, Alabdullah et al. (2022)^50^ gathered 201 dataset to develop ML method chloride migration coefficient. They used concrete aging, binder content, W/B ratio, metakaolin, sand, coarse aggregate, and concrete compressive strength as input features. Their model revealed that concrete compressive strength is the most critical parameter in predicting the chloride migration coefficient. However, they found that aggregate content showed the minimum importance. Also, it was deduced from their ML model that using metakaolin has a vital influence on chloride penetration resistance with the optimum dosage of 15%. In this regard, Amin et al. (2022)^51^ used the same dataset and input features of metakaolin-based concrete to develop a GEP model of predicting RCPT. Their model showed that the concrete age is the most noteworthy factor, along with aggregate content.

**Table 2:** Artificial intelligence (AI) techniques used in the literature for predicting concrete chloride resistance.

Research	AI method	Input number	Input variables	Concrete type	Dataset number
Parichatprecha & Nimityongskul (2009)^30^	ANN	8	W/B, cement, FA, SF, water, SP, sand, coarse	HPC	86
Song & Kwon (2009)^31^	ANN	8	W/B, cement, FA, SF, GGBFS, sand, coarse, submerged duration time	HPC	120
Hodhod & Ahmed (2013)^32^	ANN	5	W/B, cement, FA, GGBFS, curing age	HPC	300
Ghafoori et al. (2013)^36^	Statistical & ANN models	9	W/B, cement, FA, SF, sand, coarse, SP, air-entraining admixture, VMA	SCC	24
Inthata et al. (2013)^40^	Statistical & ANN models	6	W/B, percent replacement, testing ages, pozzolans types, aggregate to cement ratio,${f}_{c}$	NC & HSC with pozzolans	270
Boğa et al. (2013)^41^	ANN & neuro-fuzzy	4	Cure type, curing period, GGBFS, CNI ratio	GGBFS & CNI Concrete	162
Marks et al. (2015)^42^	ML	4	Cement, FA, water, specific surface of FA	HCFA	56
Slika & Saad (2016)^43^	Kalman Filter	4	Initial Surface Chloride Concentration, Constant for surface chloride build-up, Reference Diffusion Rate, Age	NC	Limited local data
Asghshahr et al. (2016)^44^	ANN & CART	4	W/B, environmental condition, penetration depth, SF	SF Concrete	162
Hoang et al. (2017)^45^	ML models with MGGP & MARS	4	Mortar age, depth of measured point, diffusion dimension, the presence of reinforcement	Cement Mortar	132
Mohamed et al. (2018)^37^	ANN	11	W/C, cement, GGBFS, FA, SF, water, SP, sand, coarse, age, charge	SCC	86
Gao et al. (2019)^46^	GP	8	W/C, temperature, relative humidity, concrete cover, load presented by the burial depth, chloride-ion binding capability, degradation coefficient of the structure, synthesized coefficient of the chloride-ion content	NC	25
Najimi et al. (2019)^38^	ANN	8	W/B, cement, FA, SF, sand, coarse, AEA, SP	SCC	72
Delgado et al. (2020)^52^	ANN	7	W/C, Cement type, water, addition type, curing period, days, number of drying/ wetting cycles	No limitation	243
Kumar et al. (2020)^39^	MARS & MPMR	3	FA, SF, temperature	SCC	360
Cai et al. (2020)^53^	ML	12	Cement, FA, GGBFS, SF, SP, water, sand, coarse, exposure time, temperature, chloride content in seawater, exposure type	No limitation	642
Yao et al. (2021)^54^	PSO on BP neural network	8	W/B, cement, GGBFS, FA, SF, sand, coarse, duration time	No limitation	120
Mohamed et al. (2021)^33^	ANN	10	W/B, concrete age, cement, GGBFS, FA, SF, water, SP, sand, coarse	SCC	294
Ahmad et al. (2021)^47^	GEP	11	Cement, sand, coarse, SF, FA, GGBFS, SP, water, exposure time, temperature, chloride content	Waste Concrete	642
Liu et al. (2021)^4^	ANN	12	W/B, cement, GGBFS, SF, SP, coarse, sand, ${f}_{c}$, curing mechanism, testing method, exposure time, exposure environment	No limitation	653
Yuan et al. (2022)^34^	Meta-heuristic algorithm & artificial intelligence	6	Cement, FA, SF, temperature, sand, coarse	SCC	360
Taffese et al. (2022)^55^	ML	17	W/B, cement type, cement, slag, FA, SF, lime filler, sand, coarse, total aggregates, SP, AEA, slump test, slump flow, air content, density,${f}_{c}$	No limitation	Model I:134 Model II:131 Model III:176 Model IV:91
Tran et al. (2022)^56^	ML	6	W/B, exposure condition, cement, SF, time exposure, depth of measurement	No limitation	404
Golafshani et al. (2022)^8^	Metaheuristic artificial intelligence	9	Cement, water, SF, coarse, sand, SP, curing mechanism, exposure time, the exposure condition	No limitation	216
Ge et al. (2022)^35^	ML	6	W/C, cement, FA, SF, a ratio of coarse and fine aggregates, temperature	SCC	360
Tran et al. (2022)^57^	ML	11	Cement, FA, GGBFS, SF, SP, water, sand, coarse, temperature, chloride concentration in seawater, exposure time	No limitation	386
Liu et al. (2022)^48^	ML	9	Cement, water, sand, coarse aggregate, recycled coarse, FA, GGBFS, aggregate water absorption, curing age	RAC	226
Tran (2022)^58^	ML	9	W/B, C_3_A content, cement, FA, GGBFS, SF, aggregate, water, specific surface of FA	SCMs Concrete	127
Guo et al. (2022)^59^	ML	6	W/B, cement, FA, GGBFS, exposure environment, exposure time	No limitation	366
Guo et al. (2022)^60^	Fuzzy logic	7	W/B, cement, FA, GGBFS, temperature, exposed marine environment, duration of exposure	No limitation	495
Taffese & Espinosa-Leal (2022)^61^	ML	15	W/B, Cement types, water, cement, slag, FA, SF, lime filler, sand, coarse, total aggregate, plasticizer, SP, AEA, concrete age at migration test	No limitation	843
Alabdullah et al. (2022)^50^	ML	7	W/B, aging of concrete, binder content, MK, sand, coarse,${f}_{c}$	MK HSC	201
Amin et al. (2022)^51^	GEP	7	W/B, age of the sample, amount of binder, sand, coarse, MK,${f}_{c}$	MK Concrete	201
Jin et al. (2022)^49^	ANN	9	W/C, cement, water, coarse aggregate, sand, SP, recycled aggregate replacement rate, water absorption rate of coarse aggregate, apparent density of coarse aggregate	RAC	112

Regarding the second category (models with no limitations), many studies gathered many types of concrete data in a specific database^47,52–54^. For instance, Delgado et al. (2020)^52^ used 243 datasets without concrete type limitations to develop ANN models of chloride depth penetration and diffusion coefficient. They considered cement type as an additional input variable for the first time. Based on their model, curing time is the most important input feature for the chloride penetration ANN model. In this field, Cai et al. (2020)^53^ collected 642 datasets for different types of concrete to predict surface chloride concentration using the ensemble ML method. Twelve input variables were considered in their model, including cement, water, FA, GGBFS, SF, Superplasticizer, fine aggregate, coarse aggregate, exposure time, Annual mean temperature, chloride content in seawater, and exposure type. Their predicting model showed that the exposure condition (i.e., tidal, splash and submerged zones) and W/B ratio seem to be the greatest important factors affecting the surface chloride concentration. Ahmad et al. (2021)^47^ gathered a dataset of concrete containing waste material to predict the surface chloride concentrations using gene expression programming (GEP). According to these studies, Liu et al. (2021)^4^ collected 653 datasets of different concrete types to develop an ANN model for predicting the chloride diffusion coefficient. They also considered the concrete compressive strength, curing mechanism, and common input variables. Tran et al. (2022)^57^ used an ensemble decision tree boosted (EDT Boosted) model to predict the surface chloride concentration considering the 386 datasets. Their model indicated that the fine aggregate content is the key parameter influencing the Cs. Tran (2022)^58^ concentrated on 127 concrete data-containing SCMs to develop an ML model for the chloride diffusion coefficient. They ignored using temperature and curing time as input parameters. It can be deduced from their model that water content and FA dosage have the most and least effect on chloride diffusion coefficient precision, respectively. They reported that GGBFS content has also a low impact on the output. Based on their model, water content and W/B ratio has negative relationship with the chloride diffusion coefficient. Guo et al. (2022)^59,60^ developed ML and Fuzzy logic system methods on two datasets (366 and 495 numbers) to predict surface chloride concentration without considering a specific type of concrete. Their models indicated that by increasing the W/B ratio, Cs increases. Moreover, mineral admixture and the W/B ratio affect the surface chloride concentration. Based on their model, FA and GGBFS cause higher and lower surface chloride concentration, respectively. Finally, the most recently studied in this field was conducted by Taffese & Espinosa-Leal (2022)^55^, where the ML method was used to predict the migration coefficient of different types of concrete in a unique dataset. Although they gathered an extensive experimental database, four separate models were used for different input variable groups due to some missing input variables. Each group has less than 200 datasets, including (1) first group with 134 dataset containing W/B ratio, Cement, Slag, FA, SF, fine aggregate, coarse aggregate, superplasticizer, migration test age, and cement types; (2) the second group uses 131 datasets with the same as group first input variables along with fresh density; (3) third group has 176 datasets considering the same input variables of the first group and additional parameters of compressive strength test age and compressive strength; and finally, (4) fourth group having all input variables with 91 datasets, showing that a limited number of the dataset mentioned both concrete compressive strength and fresh density in their studies. Hence, they couldn’t use the ML method on all 834 datasets due to the huge amount of missing input data. Similarly, Taffese & Espinosa-Leal (2022)^61^ only used the final 204 datasets for the ML method, considering input variables of W/B ratio, cement, water, slag, FA, SF, fine aggregate, coarse aggregate, total aggregate, superplasticizer, and air-entraining agent, and migration test age. Their finding from the proposed model showed that binder content and aggregate content considerably affect the predicting model. They also found that cement type has no impact, which is considered a weak predictor for classifying concrete chloride resistance and can be removed from the model. In this filed, Tran et al. (2022)^56^ used the ML technique to predict chloride content based on 404 dataset. Based on their model, exposure condition and depth of measurement are the most important parameters for the prediction of chloride content. In this context, Golafshani et al. (2022)^8^ developed a marine creatures-based metaheuristic artificial intelligence model to predict the apparent chloride diffusion using 216 dataset. Their model indicated that the exposure time and curing conditions considerably control the performance of the predicting model.

One of the main criteria for evaluating the performance of an AI model is simplicity. Accordingly, the number of input variables and accessibility should be considered in a predicting model. Hence, based on the experience of other proposed models and SHapley Additive exPlanations (SHAP) results, some input variables can be ignored to predict the chloride diffusion coefficient. Predominant input variables reported for each literature on AI methods are summarized in Table 3. It is clearly shown that based on the number of datasets considered, different parameters found by the AI methods have the most influence on predicting models of chloride resistance. Water, cement, W/B ratio, SCMs, chemical admixtures, aggregate content, and exposure conditions were severally considered by the literature as input variables in AI methods. However, there are differences of opinion about whether or not to consider some parameters. For instance, regarding cement type, although Delgado et al. (2020)^52^ reported the critical role as input illustrative in predicting chloride penetration in concrete, Taffese et al. (2022)^55,61^ showed its lack of influence as a predictor.

**Table 3:** Most vital features reported by AI models for predicting concrete chloride resistance.

Reference	Order of important predicators		
	1	2	3
Parichatprecha & Nimityongskul (2009)^30^	Cement content	FA	SF
Ghafoori et al. (2013)^36^	SCMs content	W/B ratio	Aggregate content
Inthata et al. (2013)^40^	Ground rice husk ash	FA	Ground bottom ash
Asghshahr et al. (2016)^44^	Penetration depth	–	–
Delgado et al. (2020)^52^	Curing time	Cement type	Addition type
Cai et al. (2020)^53^	Exposure condition	W/B ratio	Binder content
Yuan et al. (2022)^34^	Temperature	SF	–
Taffese et al. (2022)^55^	W/B ratio	Coarse aggregate	Water
Tran et al. (2022)^56^	Exposure condition	Measurement depth	W/B ratio
Golafshani et al. (2022)^8^	Exposure time	Curing conditions	Exposure condition
Ge et al. (2022)^35^	Temperature	–	–
Tran et al. (2022)^57^	Sand content	Chloride concentration in seawater	Exposure time
Liu et al. (2022)^48^	Water content	GGBS content	Cement content
Tran (2022)^58^	Water content	Aggregate content	C_3_A content
Taffese & Espinosa-Leal (2022)^61^	Binder content	Aggregate content	W/B ratio
Alabdullah et al. (2022)^50^	Compressive strength	Aging	W/B ratio
Amin et al. (2022)^51^	Concrete age	Aggregate content	W/B ratio
Jin et al. (2022)^49^	Sand content	Recycled aggregate content	–

Moreover, the greatest numbers of the dataset did not precisely mention the cement type in the experimental program^49^. Accordingly, most developed AI models ignored this variable as an input feature. Also, only a few studies used concrete compressive strength as an input variable in AI methods^4,40,50,51^. Similarly, only Taffese et al. (2022)^55^ considered concrete density as one of the input variables to predict chloride resistance. Most studies also ignored the temperature, while many models considered it an input feature^34,35,39,46,47,53,57,60^. It may be due to the missing data in the experimental research. As mentioned in Table 3, there is no clear trend for the most important predictor in the models presented by the literature, so different input variables were reported. However, it can be deduced from Table 3 that parameters of cement content, SCMs content (FA and SF), curing time, and aggregate content (sand and coarse) should be considered for predicting models of concrete chloride resistance. Although other parameters of temperature, penetration depth, exposure time, and exposure condition were also found as vital factors affecting the predicting models, they cannot be considered definite input parameters due to the lack of data in the experimental dataset. The conflicting trend obtained by the previous predicting AI models (Table 3) can be attributed to some critical reasons, including (1) the number of the dataset collected in each model; (2) the types of concrete considered; (3) input variables selected for each model; (4) AI methods used to obtain a predicting model; and (5) existence of missing input data in some experimental program. Hence, although the literature presented valuable predicting models, they may not be well-suited to be considered reliable chloride resistance predicting models for all experimental databases considering different types of concrete.

### Research significance

As reviewed in this section, introducing a unique model for each concrete type is impractical. Accordingly, a significant research gap should be filled by developing a novel AI method using all available datasets. Additionally, due to the lack of consistency and exitance of missing data in the details of mixtures tested by the literature, most of the previous predicting AI models ignored these valuable experimental datasets in their model, causing a significant reduction in the accuracy and comprehensiveness of the existing predicting models for all types of concrete. Accordingly, the present study uses the ANN method as a dataset arranging technique to practically predict missing details in datasets for the ML models. In other words, the ANN method helps to prepare an accurate and efficient dataset for ML input and prevents the deletion of valuable data. Additionally, prior studies have examined the forecasting of concrete's chloride migration coefficient, but there has been a lack of research on the integration of regression and classification tasks. Moreover, there is a scarcity of scholarly investigations pertaining to the advancement of artificial neural networks and machine learning techniques for the purpose of constructing a robust predictive model using a comprehensive dataset comprising over 1000 data points. Therefore, it is imperative to address a notable deficiency in research by devising an innovative artificial intelligence (AI) approach that effectively utilizes all accessible datasets. Hence, the present study intends to address this issue by following the objectives:

1. Developing a unique ML method to predict the chloride diffusion coefficient for all types of concrete.
2. Introducing a developed dataset-cleaning technique (DCT) to cover the missed database of all experimental works using ANN.
3. Compare classification algorithms with regression ones in the supervised learning method.
4. Finding the most and least influencing parameters controlling the predicting model for chloride diffusion coefficient.

**Figure 1:**
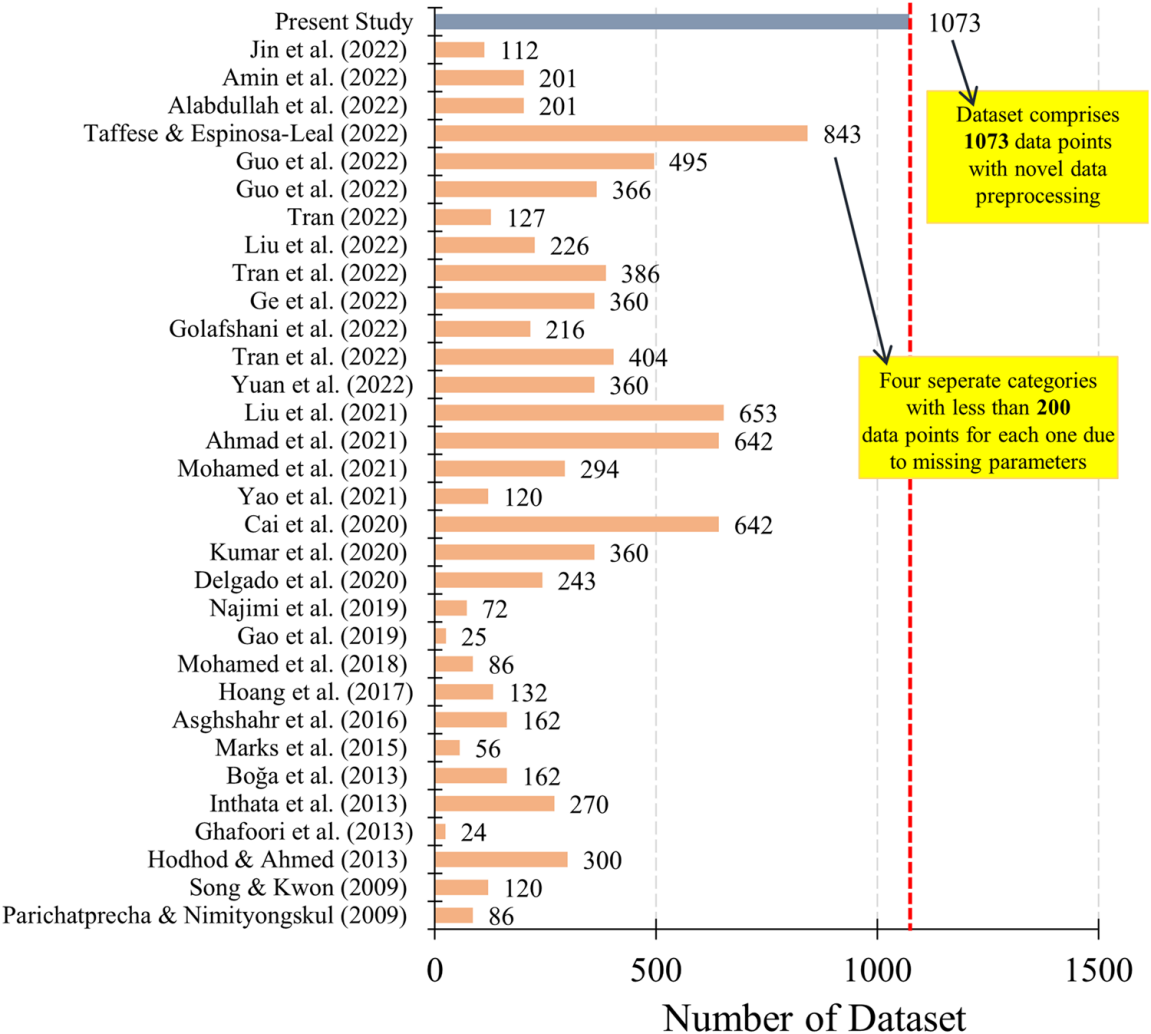
Number of datasets used in the literature as compared to the present study.

To achieve these objectives, a comprehensive experimental database containing 1037 datasets was gathered in the present study to predict the ${D}_{nssm}$ using 12 various features of water content, cement, slag, FA, SF, fine aggregate, coarse aggregate, superplasticizer, fresh density, compressive strength test age, compressive strength, and migration test age (Fig. 1). Due to the missing data for fresh density and compressive strength, previous studies couldn’t practically use all dataset in a unique model, so that ML method applied on four separate groups. However, the present study intends to solve this issue using the ANN method to predict the missing data through a precise approach. Linear Regression (such as elastic net, lasso, Ridge), decision tree, random forest, boosting algorithms, support vector machine, and k-nearest neighbors algorithm were considered in the present study for regression method. Regarding the classification method, different algorithms were also selected for predicting ${D}_{nssm}$, including support vector machine, random forest, lightGBM, XGBoost, Logistic Regression, k-nearest neighbors (KNN), and decision tree.

## Machine learning method (ML)

Generally, the ML method is the branch of AI that deals with the application of algorithms that allow computers to advance patterns using the experimental database. The main intention of the ML method is to automatically learn to identify complicated patterns (relations between variables) and then make ingenious judgments based on datasets. The dataset is a set of logical (laboratory) records with unique characteristics called machine input data or features. Each of these input data is also called a predictor. Moreover, the intelligent decision that the machine should make after learning this data is considered a prediction model for a particular output (or target). In other words, the machine has reached intellectual maturity by finding a logical connection between the input data and the results. It provides a logical model of a real physical phenomenon. The main problem exists where the set of all possible behaviors given all potential inputs is too enormous to be covered by the set of observed examples (training data). Accordingly, the learner should generalize from the training data to produce expedient target data for new conditions out of the datasets. Pattern recognition, commonly accompanied by classification, is the most popular use case for the ML method. Although the quantity and quality of datasets play a significant role in the training process, selecting appropriate features or input variables (unique dataset characteristics) affects the ML model's performance considerably.

**Figure 2:**
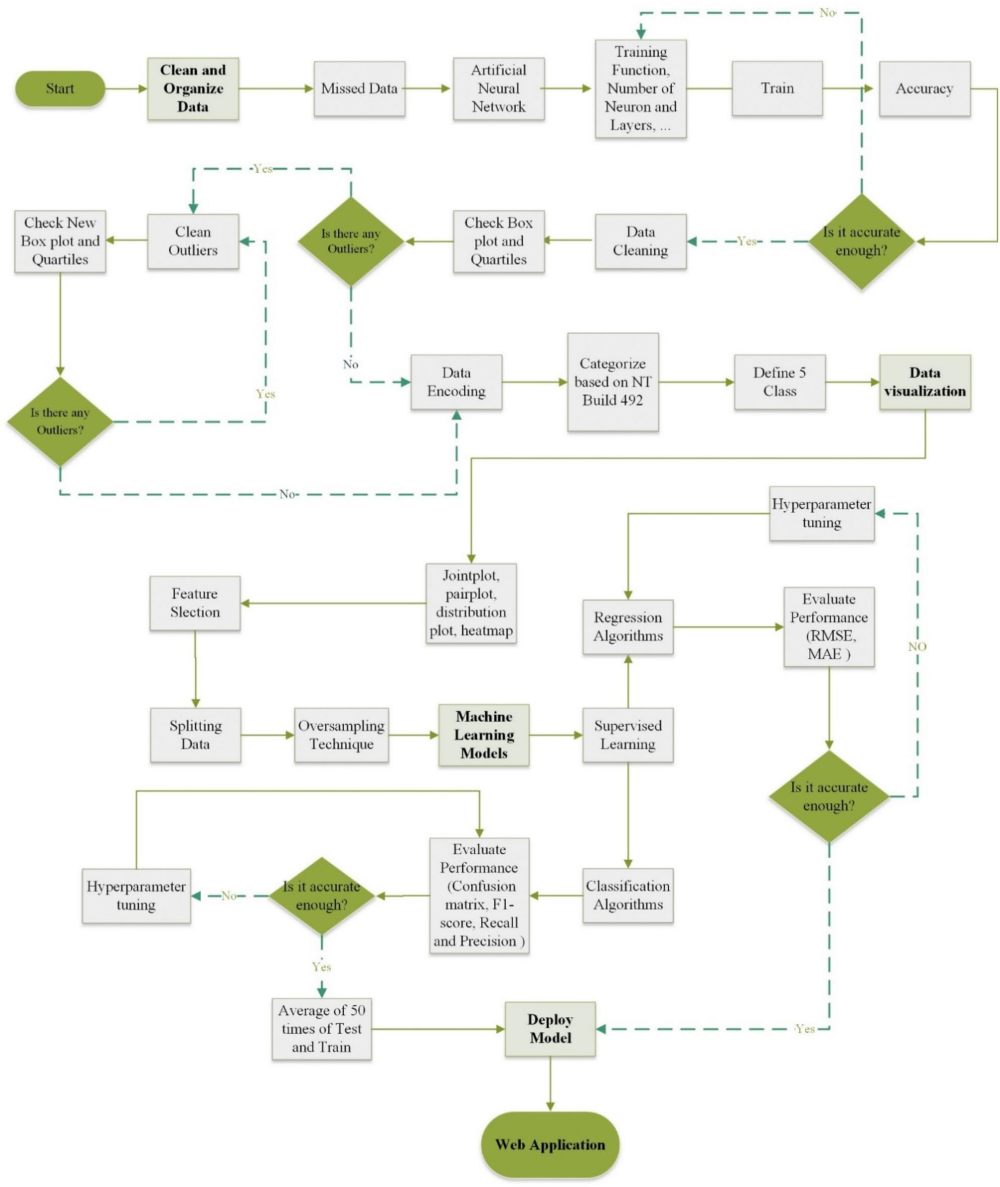
Flowchart presenting the methodology of the machine learning (ML) technique proposed in the present study.

The present study comprehensively assesses the potential of using regression and classification algorithms in the ML method to predict the non-steady-state migration coefficients (${D}_{nssm}$) without any limitation for the concrete type or missing input data. Using such a substantial experimental database (1073 datasets) in the ML method is critical to achieving a unified model. A practical DCT is used in the present study to compensate for the missing data in the literature. The figures and machine learning models utilized in this study were implemented through the utilization of the Python programming language^62^. The ANN model in Figure S1 was generated using MATLAB (2019b)^63^ in this study. The flowchart of the ML methodology proposed in the present study is shown in Fig. 2. The ML method proposed in the present study consists of three main steps, including (1) data cleaning technique; (2) data visualization; and (3) ML models. Each of these steps is explained in the following subsections.

### Dataset-cleaning technique (DCT)

**Figure 3:**
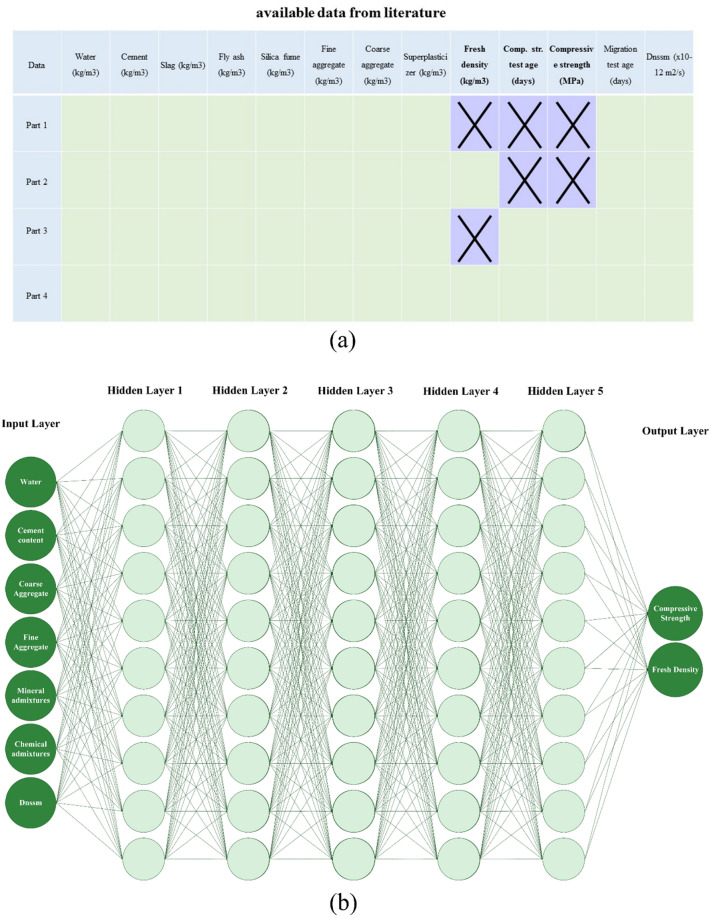
Dataset-cleaning technique (DCT) procedure: (**a**) different categories of literature regarding missing data; (**b**) Using the feed-forward back propagation network and the Levenberg–Marquardt training function for reproducing missing data.

Experimental datasets used in the present novel ML models after considering the outlier’s removal are summarized in Table 4. Totally 24 research papers containing 1073 datasets were collected in the present study. As shown in Fig. 3a, experimental databases gathered have three missing input features for some datasets, including fresh density, compressive strength test age, and concrete compressive strength. To address this issue, Taffese et al. (2022)^55^ divided the datasets into four separate groups with an experimental database lower than 200 datasets. However, this method cannot be efficient as the ML method is considerably affected by the quality and quantity of the dataset. Moreover, achieving a reliable unified model using all datasets is required. Accordingly, the feed-forward back propagation network and the Levenberg–Marquardt training function were used in the present study to predict the missing data using different hidden layers for each dataset group based on the missing parameter type (Fig. 3b). The accuracy of the ANN prediction is illustrated in the supplementary file (Figure S1). For this ANN method, water-to-binder (W/B) ratio, cement content, aggregate content, mineral admixtures, aggregate content, chemical admixtures, and ${D}_{nssm}$ were considered as input layers to predict the missing values for the concrete compressive strength and fresh density. Regarding this high and adequate accuracy of the ANN method, the problem of the missing dataset was entirely solved, and accordingly, the complete datasets were used in the ML method.

**Table 4:** Experimental datasets used in the present novel ML models after considering outlier’s removal.

Reference	Count	W/B (–)	Cement (kg/m^3^)	Slag (kg/m^3^)	FA (kg/m^3^)	SF (kg/m^3^)	Fine aggregate (kg/m^3^)	Coarse aggregate (kg/m^3^)	SP (% by binder wt.)	Fresh density (kg/m^3^)	${f}_{c}$ (MPa)	${D}_{nssm}$
Costa & Appleton (1999)^64^	24	0.30–0.50	300–500	0	0	0–21.5	0–822	1131–1704	0–0.02	** × **	34–66	0.88–4.83
Thomas & Bamforth (1999)^65^	18	0.48–0.66	110–288	0–255	0–98	0	585–660	1240–1305	0	** × **	37.9–49.6	0.59–10
Hao–bo & Guo–zhi (2004)^66^	16	0.36	192–480	0–147	0–216	0–24	614–696	1061–1147	0.9–1.0	** × **	49.1–63.1	0.74–7.0
Alizadeh et al. (2008)^67^	2	0.5	370–400	0	0	0–30	778	956	0–0.01	2355–2370	39–54.1	0.99–1.51
Song & Kwon (2009)^31^	120	0.37–0.47	178–454	0–227	0–136	0–23	745–838	921–976	0.01	** × **	** × **	0.22–6.3
Shekarchi et al. (2009)^68^	60	0.35–0.50	350–400	0	0	0–50	778–936	955–1022	0–0.02	2255–2467	33.9–79.6	0.22–12.54
Audenaert & De Schutter (2010)^69^	20	0.37–0.60	300–450	0	0–240	0	626–923	583–1225	0	** × **	40.3–74.7	0.15–13.5
Jain & Neithalath (2011)^70^	15	0.40	344–430	0	0–86	0–39	730–743	1050–1058	0	** × **	** × **	1.65–9.94
Liu et al. (2011)^71^	21	0.38–0.54	400–500	0	0	0	321–517	518–1098	0–0.65	1610–2360	31–75	6.5–19.1
Maes et al. (2013)^72^	4	0.50	52–350	0–295	0	0	785–791	1036–1043	0	2331–2350	** × **	3.93–8.98
Elfmarkova et al. (2015)^73^	4	0.40	312.3–520.6	0–312.3	0–442.5	0–468.5	1574.1	0	0.2–0.4	2150–2210	51.9–60.4	2.49–11.15
Real et al. (2015)^74^	86	0.35–0.55	175–450	0	0–180	0–45	703–1272.7	0–593.2	0	** × **	16.9–84.2	3.8–22.8
Bogas & Gomes (2015)^75^	49	0.30–0.55	270–525	0	0–735	0–36	646–1073	266.35–915.53	0–0.83	1483–2411	30.9–76.2	3.4–19.9
Liu et al. (2015)^76^	16	0.30–0.54	144–471	0	0	0	235–874	255–1064	0–4.17	1364–2365	** × **	4.9–19.1
Farahani et al. (2015)^77^	12	0.35–0.5	350–370	0	0	30–50	782–931	955–1020	0–0.02	** × **	** × **	0.33–2.7
Park et al. (2016)^78^	105	0.35–0.55	128–470	0–282	0–141	0	669–815	927–978	0.7–1.22	** × **	** × **	1.02–42.04
Ferreira et al. (2016)^79^	15	0.45–0.60	330–440	0	0	0	600–710	1240–1240	0	** × **	18.9–44.8	5.7–54
Pilvar et al. (2016)^80^	24	0.35–0.45	297.5–400	0	0	0–60	1037–1150	692–766	0–1.10	** × **	46–80	1.41–14.3
Choi et al. (2017)^81^	12	0.40–0.60	350	0	0	0	707–742	1080–1135	0	** × **	** × **	8.74–133.6
Liu et al. (2017)^82^	15	0.38–0.53	286–454	0	0–123	0	689–729	1041–1094	0	** × **	34.6–74.3	2.3–9.27
Shiu & Yang (2020)^83^	16	0.34– 0.65	168–495	0– 242	0	0	786	934	0– 2.07	** × **	231– 483	3.24– 26.7
Naito et al. (2020)^84^	138	0.19–0.65	13.02–2384.97	0–1284.4	0–74.2	0–29.7	27.5–904.9	26.4–1187.2	0	2176.9–2447.6	19.2–80.6	0.55–30.6
Sell Junior et al. (2021)^85^	6	0.45–0.65	320–400	0	0	80	587.2–640.0	880.8– 960.0	0	** × **	40.6– 74.5	0.20– 6.02
Pontes et al. (2021)^86^	20	0.35– 0.55	245–450	0	0– 135	0– 27	606.2– 822.2	935.1– 1154.96	0	** × **	26.1– 78.0	5.9– 25.2

### Data preprocessing

**Figure 4:**
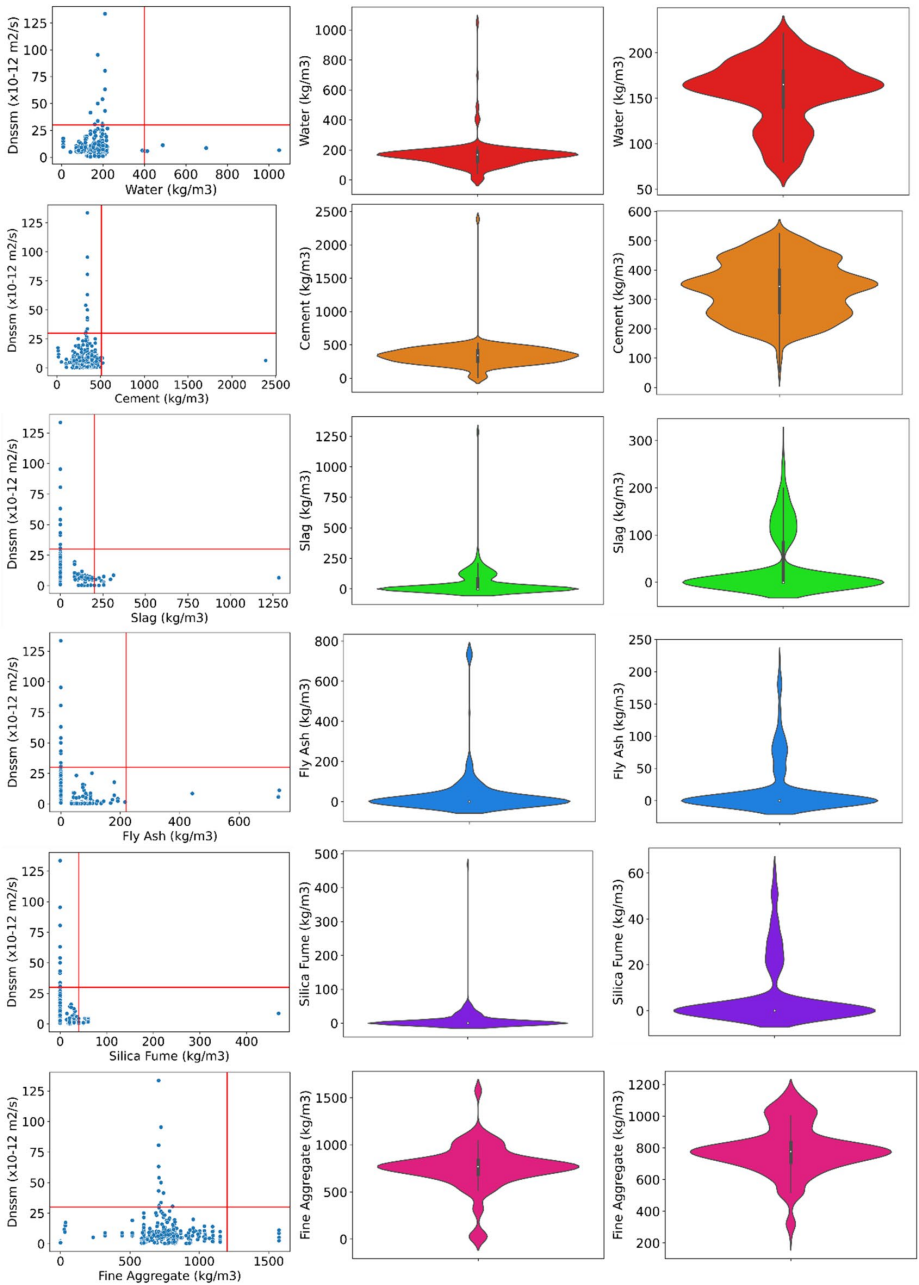

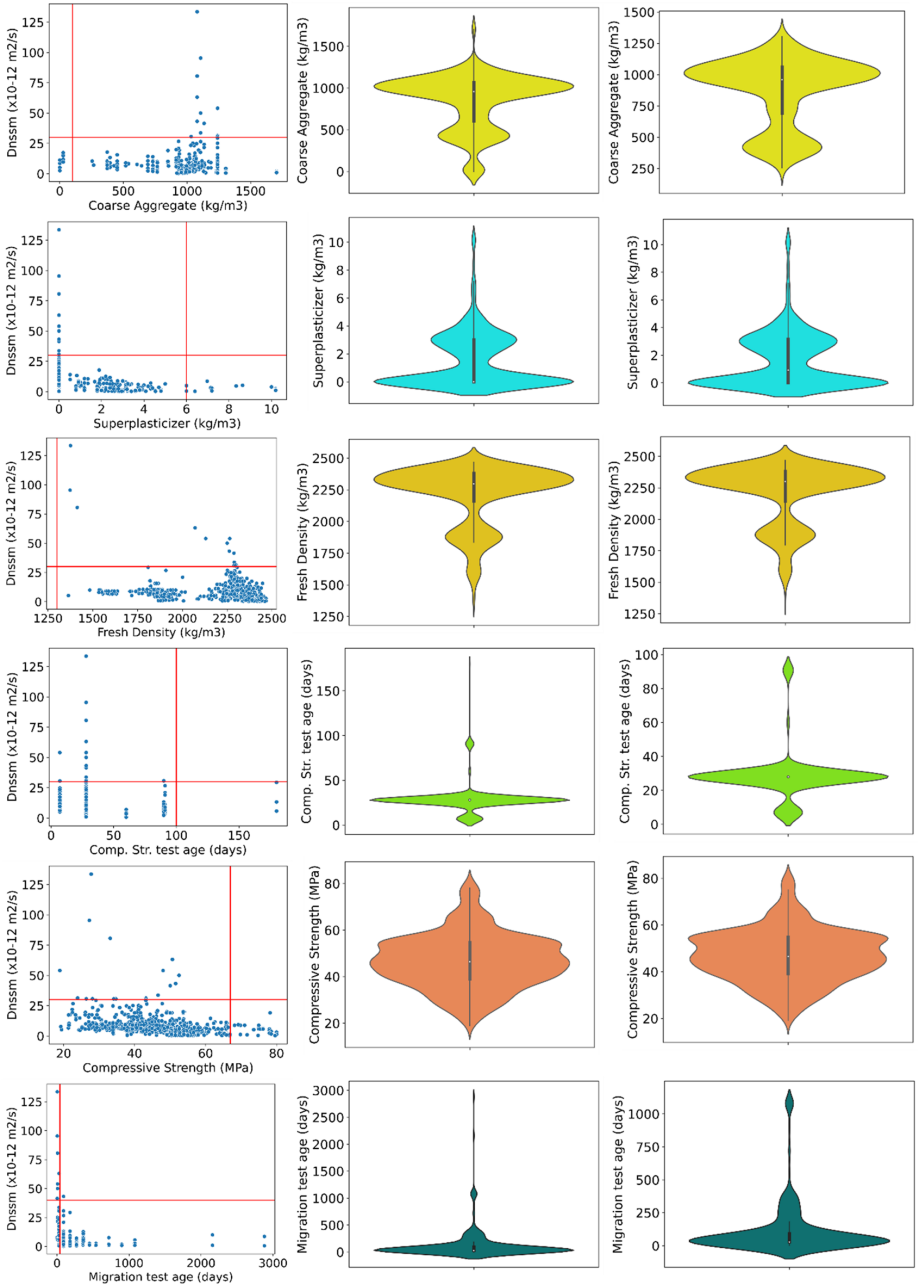
The procedure of outlier’s detection (left and middle ones show the dataset with outliers, right one represents the dataset without outliers).

Generally, outliers are a few parts of datasets that are meaningfully different from the rest of the database. They are usually anomalous observations that deviate from the data distribution and are commonly caused by inconsistent data entry or inaccurate observations. The reason behind removing each outlier is necessary. One of the main methods to consider the removal of outlier datasets is to analyze with and without these skewed datasets and explain the differences. Descriptive statistics of the datasets considered in the present study before removing the outliers are summarized in Table 5. Regarding the output parameter (${D}_{nssm}$), a range of 0.22 to 133.6 (× 10^−12^) m^2^/s was gathered in the present study. However, only a few contents of datasets are in the range of ${D}_{nssm}>50$, and accordingly considered as outliers. Another analysis of the outlier’s detection is shown in Fig. 4 for each input variable. For superplasticizer (SP) dosage, SP values higher than 6 kg/m^3^ were removed from the datasets. A range of 200–1500 kg/m^3^ was kept as the main database of fine aggregates, while experimental databases out of this range were considered outliers (Fig. 4). Concrete compressive strength of higher than 67 MPa was removed due to the high concentration of datasets for ${f}_{c}\le 67$ MPa. Statistical analysis confirmed that the content of SF higher than 40 kg/m^3^ should be removed as outliers. Most of the datasets were in the range of 2000 days for migration test age, so that higher than this period are considered outliers (Fig. 4). Only 1 dataset of all experimental databases has slag content higher than 400 kg/m^3^, and accordingly removed from the ML analysis as an outlier data. Similarly, only one dataset has cement content higher than 510 kg/m^3^, which was removed from the ML method. Few datasets were selected as outliers for FA higher than 400 kg/m^3^. Although most experimental databases tested concrete compressive strength at a test age lower than 100 days, three tested the sample’s compressive strength more than 175 days’ measurement and were removed as outliers. Experimental datasets of coarse aggregate out of the 200–1300 kg/m^3^ range were selected as outliers (Fig. 4). For water content, this inliers range is about 50–250 kg/m^3^. However, no dataset was removed as an outlier data for fresh density of concrete samples. Analysis of the dataset for each input parameter before and after removing outliers is also illustrated in Fig. 4. Finally, a total amount of 965 datasets were obtained after removing outliers. As mentioned in Table 1, after checking the data preprocessing, the output parameter of ${D}_{nssm}$ was divided into five categories with data encoding ranging from 0 to 4.0 based on the recommendation of NT Build 492^13^ so that a higher amount of data encoding shows the higher chloride penetration resistance of concrete mixture with negligible chloride ion penetrability.

**Table 5:** Descriptive statistics of the input and target variables used in the ML models before outlier removal consideration.

	Variables	Count	Mean	Min	Max	Std. Dev	25%	50%	75%
Input Parameters	Water (kg/m^3^)	1073	165.15	8.46	1049.39	96.00	122.50	168.00	189.00
	Cement (kg/m^3^)	1073	340.23	13.02	2384.97	189.34	250.96	344.10	414.00
	Slag (kg/m^3^)	1073	42.40	0.00	1284.44	113.99	0.00	0.00	83.65
	Fly ash (kg/m^3^)	1073	34.79	0.00	735.00	116.06	0.00	0.00	0.00
	Silica fume (kg/m^3^)	1073	7.68	0.00	468.50	31.19	0.00	0.00	0.00
	Fine aggregate (kg/m^3^)	1073	755.99	0.00	1574. 10	236.82	688.79	769.48	835.00
	Coarse aggregate (kg/m^3^)	1073	846.61	0.00	1704.00	325.52	607.00	957.00	1064.93
	Superplasticizer (kg/m^3^)	1073	1.53	0.00	10 20	1.94	0.00	0.00	3.00
	Fresh density (kg/m^3^)	1073	2211.57	1364.00	2467.00	234.15	2162.00	2297.40	2379.90
	Comp. str. test age (days)	1073	28.54	7.00	180.00	16.16	28.00	28.00	28.00
	Compressive strength (MPa)	1073	46.62	18.90	80 00	11.63	38.99	46.37	54.60
	Migration test age (days)	1073	120.07	3.00	2880.00	264.77	28 00	28.00	91.00
Output	D_nssm_ (× 10^−12^ m^2^/s)	1073	8.010	0.220	133.600	8.496	3.466	6.600	9.679

### Data visualization

**Figure 5:**
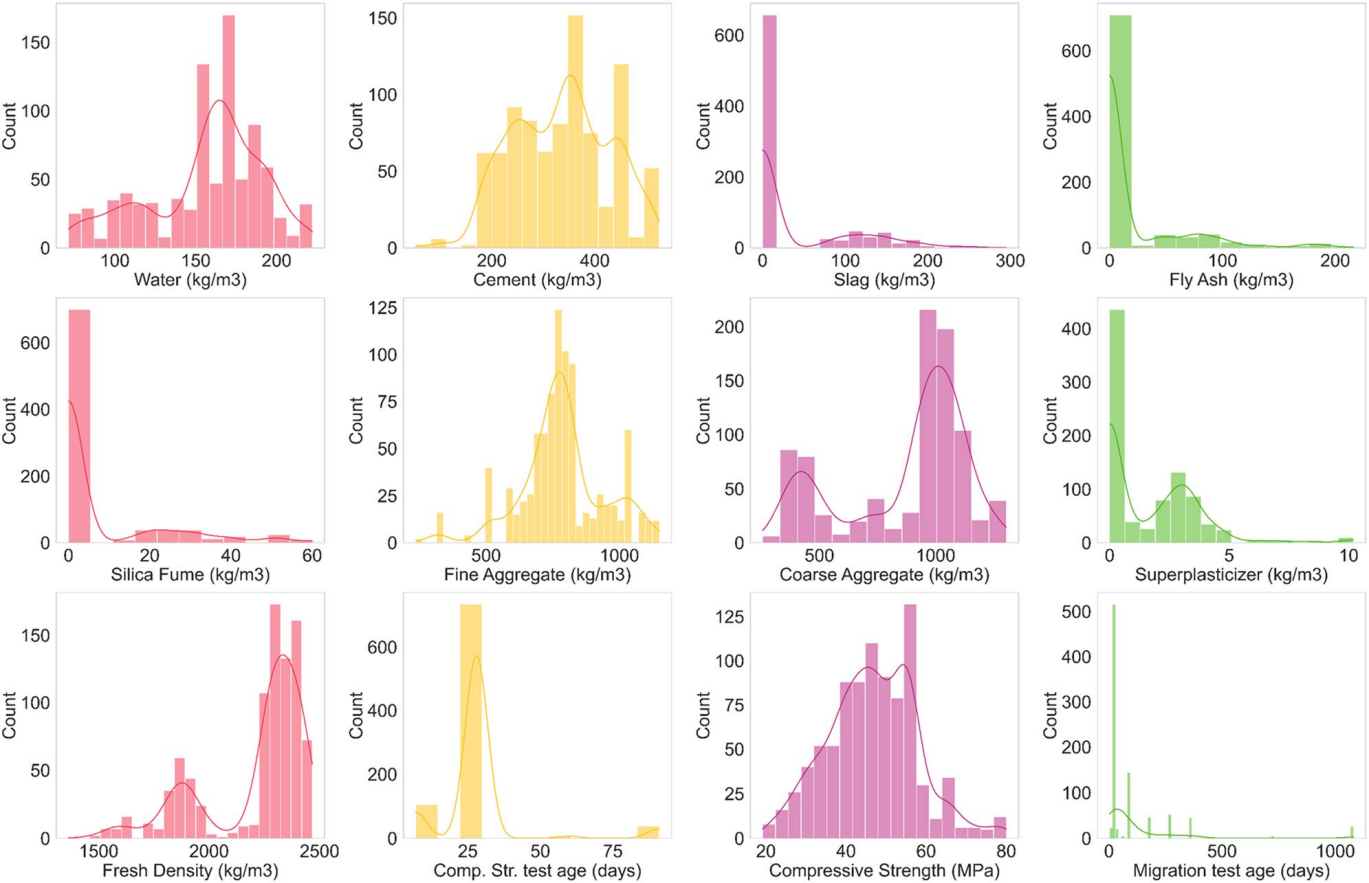
Distribution plot of variables.

To visually represent the datasets, this section utilized several plots including distplot, heatmap, and joint plot kernel density estimation (KDE), employing Seaborn, a Python library developed specifically for data visualization. Distplotm or also distribution plot is used to characterize data in histogram form. Distplotm represents a univariant set of gathered data, describing the data distribution of each variable compared to another one. As shown in Fig. 5, the distribution plot of water content shows that most of the datasets have water content in the 160–180 kg/m^3^ range. However, this domain is larger for cement content ranging from 200 kg/m^3^ to 500 kg/m^3^. The common dosages of slag, FA, and SF used in the literature to study the ${D}_{nssm}$ were 100–150 kg/m^3^, 50–100 kg/m^3^, and 20–30 kg/m^3^, respectively (Fig. 5). Regarding fine aggregates content, the literature in the field of ${D}_{nssm}$ measurement mostly used 600–1000 kg/m^3^, with the highest consumption of around 800 kg/m^3^ in concrete mixtures, while no specific range was followed for the coarse aggregate content. Most literature used 2–4 kg/m^3^ SP dosage for chemical admixture mixtures. Commonly, the datasets used in the present study are divided into two separate groups with different densities, including (1) high number, so datasets belong to the first group where concrete samples have a density of 220–2400 kg/m^3^, and (2) some portion of samples has a density ranging from 1800–2000 kg/m^3^ (Fig. 5). Also, very few samples used are lightweight concrete samples (less than 25 datasets) with fresh density lower than 1600 kg/m^3^. Most of the datasets measured the 28-day compressive strength accompanied with ${f}_{c}$ ranging from 35 to 60 MPa in the majority of samples. Finally, the distplot curve confirms that most of the concrete samples tested in the literature has ${D}_{nssm}<50$.

**Figure 6:**
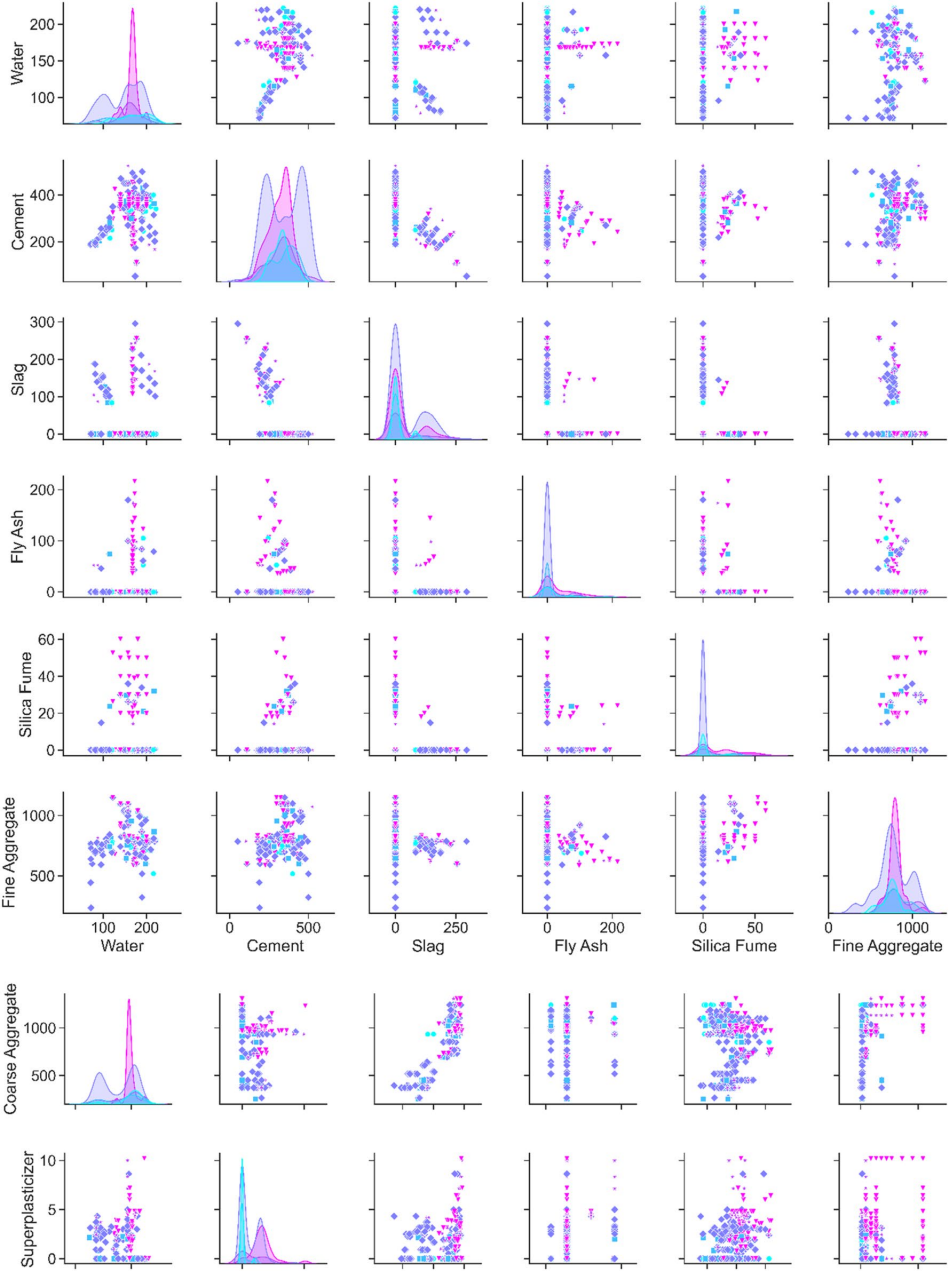

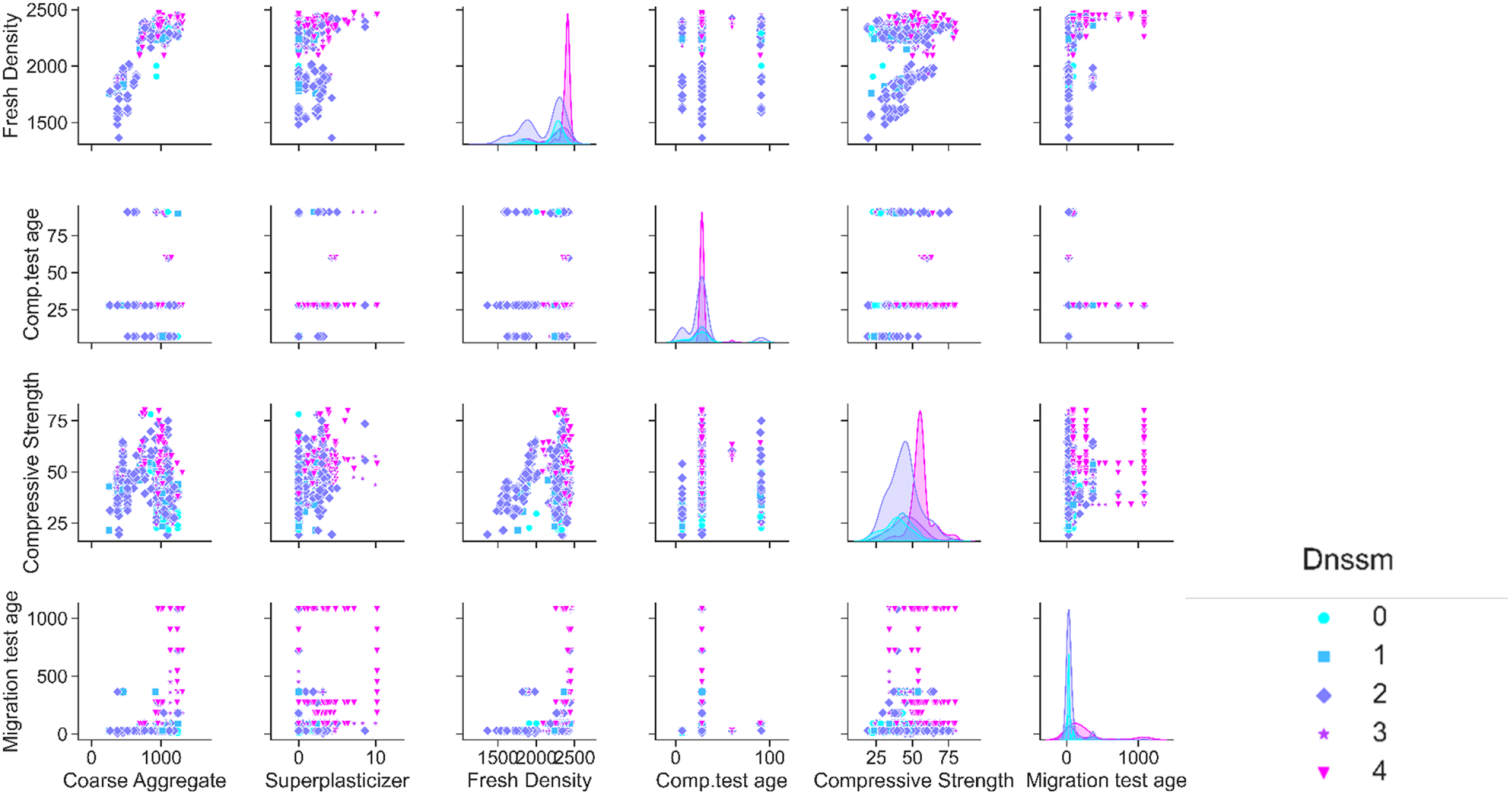
Pairplot of variables.

To illustrate the relations between variables within a dataset and reveal valuable details from the datasets, the pairplot is shown in Fig. 6. This plot provides an immaculate conception to recognize the data. The scatter plot and Kernel Density Estimation (KDE) function are shown in the pairplot so that diagonal rows are the KDEs distribution curve, representing the distribution of each parameter. However, the scatter plots presented in other cells show the relationship between variables. Different colors were also used to distinguish the output parameter classifications (${D}_{nssm}$). For instance, the water content distribution plot for four classifications (based on Table 1 data encoding) of ${D}_{nssm}$ depicted in the first row and column of the pairplot, showing that the water content distribution is near the normal distribution for ${D}_{nssm}$ classifications of 3 and 4. Regarding concrete compressive strength, all ${D}_{nssm}$ classifications show the normal distribution. Although finding appropriate justification for each parameter in contact with other variables is complicated, valuable findings can be revealed by the pairplot (Fig. 6). For instance; it can be deduced from this plot that for higher water content, samples with higher fresh density can have high chloride resistance. Also, this plot shows the appropriate relations between different powders (SCMs) so that samples containing both FA and slag show data encoding of 3 and 4 as conditions of high chloride resistance situations. A similar justification for concrete containing both FA and slag (or SF) was found. Data visualization from the pairplot also indicates that FA has a higher impact on the chloride resistance of concrete samples with high ${f}_{c}>40$ MPa. Also, FA is more efficient in reducing chloride permeability for samples containing a high content of water and a low content of cement (Fig. 6). Moreover, pairplot shows that a good relationship exists between the fine aggregate content and fresh density, so using a high content of sand along with having high density causes better chloride resistance concrete. The Pairplot curve shows that cement and SCMs contents affect the effect of coarse aggregate on the chloride resistance of concrete. Based on the data visualization results, fresh density also controls the influence of coarse aggregate on the ${D}_{nssm}$. As depicted in Fig. 6, SP content has a meaningful impact on the chloride resistance of concrete with a high W/B ratio. Coarse aggregate content, fresh density, and compressive strength similarly improve the effect of SP on ${D}_{nssm}$. Generally, results indicate that the ternary relation of SP-fresh density-compressive strength should be considered in the concrete chloride resistance. The direct effect of fresh density on the concrete compressive strength is also clearly highlighted in the pairplot, which was notably ignored by the literature regarding AI methods.

**Figure 7:**
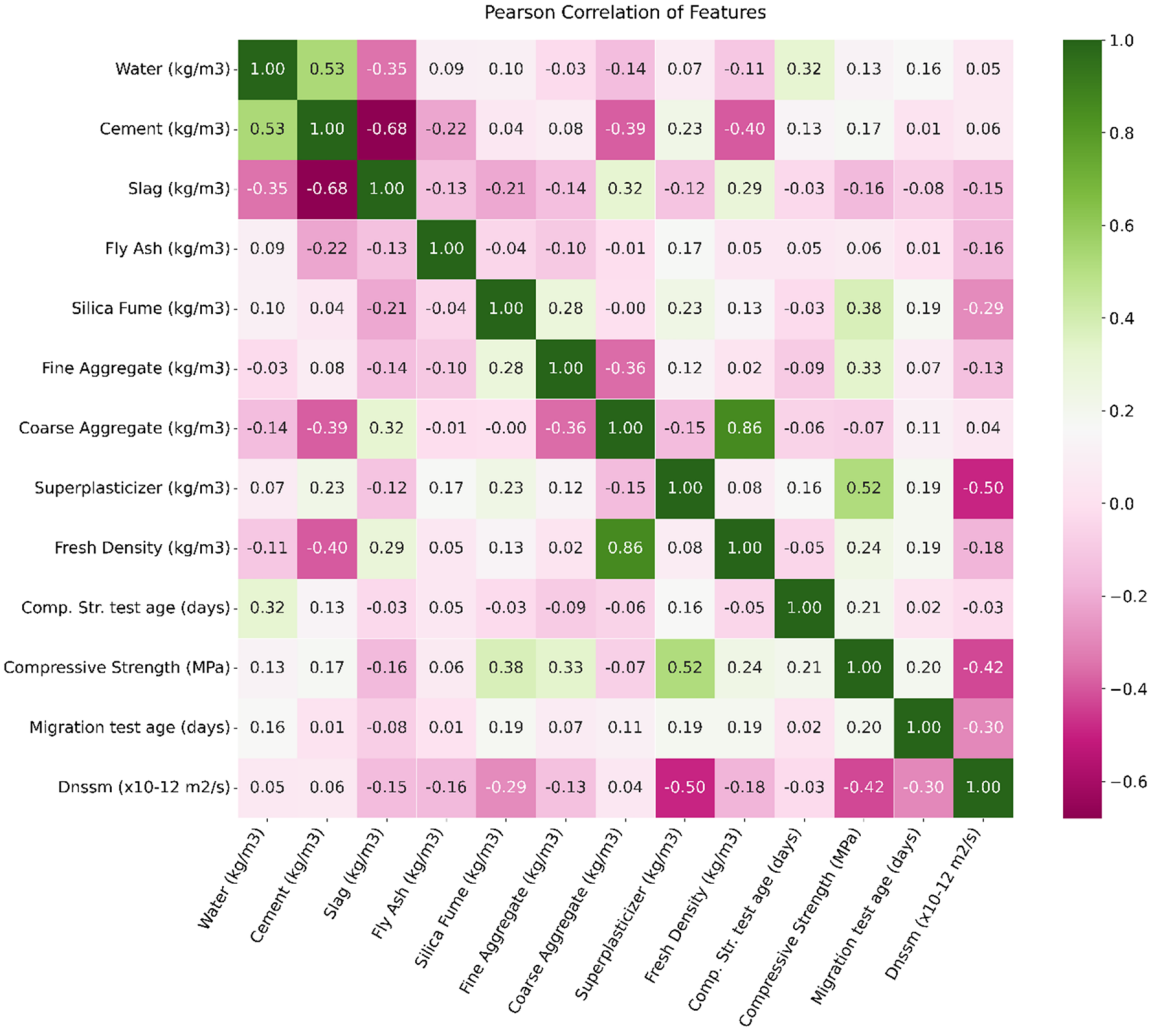
Heatmaps plot of variables.

To illustrate the relationships between two variables, the heatmap plot is shown in Fig. 7. The Pearson correlation coefficient (r) is a statistical measure used to quantify the linear relationship between two continuous variables, X and Y. It provides a numerical value that indicates the strength and direction of the linear association between the variables. The calculation of the Pearson correlation coefficient involves several steps.

For each pair of values (Xi, Yi) in the dataset, the formula subtracts the mean of X from each Xi, and the mean of Y from each Yi. This step represents the deviation of each data point from its respective mean. Then, the formula multiplies the deviations of X and Y for each data point and sums up these products across the dataset. This step captures the covariation between X and Y. The resulting sum of products is divided by the product of the standard deviations of X and Y. The standard deviation of X is calculated by summing the squared deviations of X from its mean and taking the square root. Similarly, the standard deviation of Y is obtained by summing the squared deviations of Y from its mean and taking the square root. Changing cell’s color for each axis shows the patterns in value for one or both variables ranging from -1.0, as a perfect negative linear correlation between two variables, to 1.0 representing a perfect positive linear correlation. The value of 0 designates no linear correlation between the two features. On the other hand, the heatmap plot demonstrates the independence of the variables. For instance, the first row of this plot shows that water content seems to be positively correlated with the cement content, while it is negatively correlated with the slag content. For the second raw, it can be deduced from the plot that the cement content has a strong positive correlation with the water content (+ 0.57), along with negative correlations with the slag content (− 0.68) and the fresh density (− 0.43). Also, analysis of the heatmaps plot shows that coarse aggregate content has negatively correlated with the cement content (− 0.41) and significantly has a positive correlation with the fresh density value of mixtures. This is a significant finding for the coarse aggregate content in predicting ${D}_{nssm}$. As shown in Fig. 7, SP dosage has a positive + 0.51 dependency on the concrete compressive strength. SF content, fine aggregate, and SP dosage were also found to have a notable impact on the dependency of concrete compressive strength variable.

**Figure 8:**
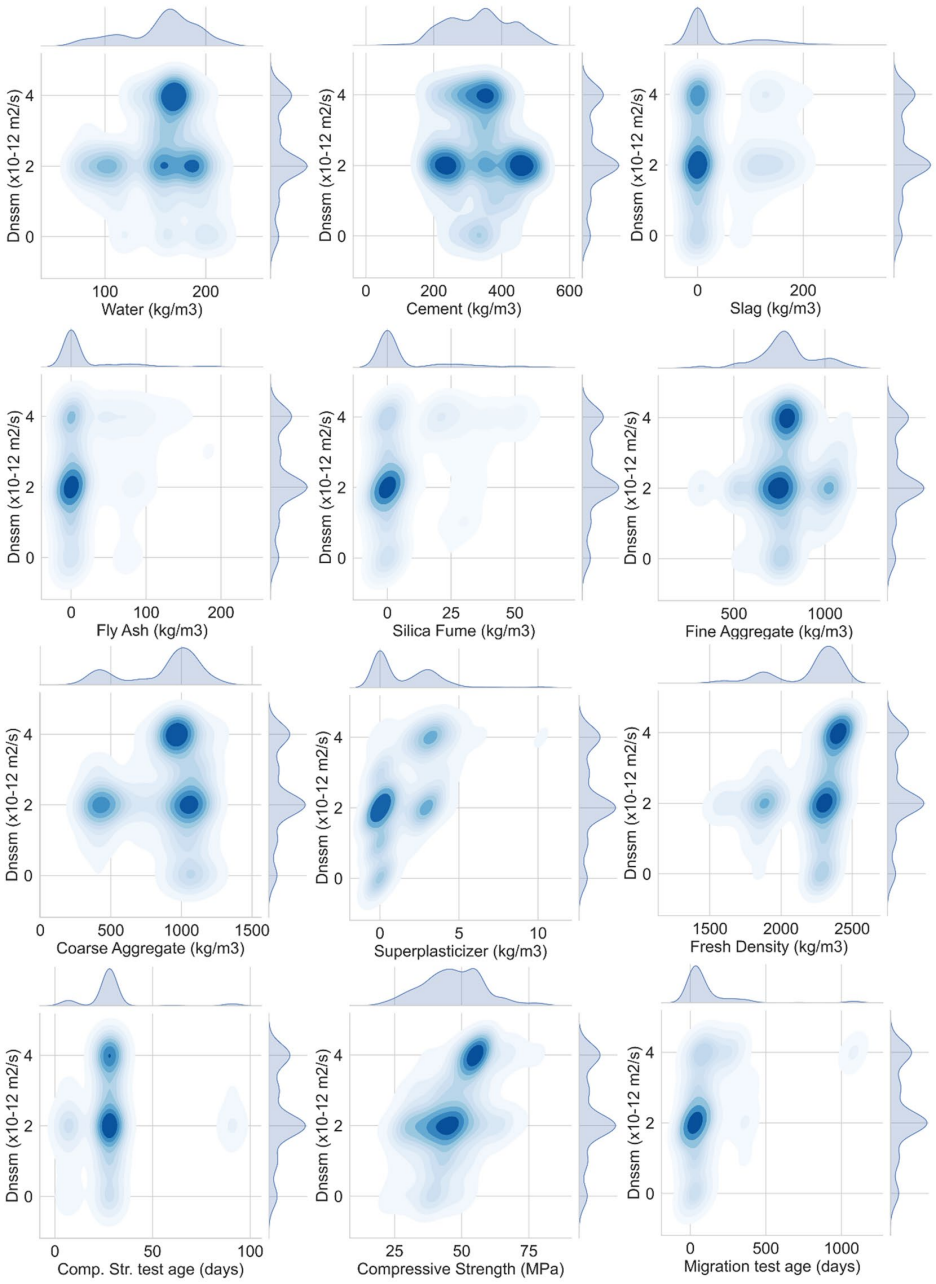
Bivariate KDE jointplot of variables (darker colors represent higher densities).

Kernel Density Estimation (KDE) jointplot for all variables against the ${D}_{nssm}$ is shown in Fig. 8. The KDE is a non-parametric approach showing the probability of the density of an independent variable. This plot contains two plots, including (1) a bivariate figure indicating how the dependent parameter (${D}_{nssm}$) changes with the variation of independent variable features; and (2) the scattering plot located at the top of the bivariate graph to display the distribution of the independent factors. KDE distribution jointplot shows that most chloride-resistant concrete mixtures with ${D}_{nssm}$ belongs to the classifications higher than 3 and has cement content ranging from 300 kg/m^3^ to 400 kg/m^3^ and a water content domain of 150–180 kg/m^3^ (Fig. 8). Among SCMs, slag content (maximum 50 kg/m^3^) shows better correlation as compared to FA and SF. Moreover, it can be deduced from the KDE jointplot that highly resistant concrete mixtures have fine aggregate and coarse range of 700–850 kg/m^3^ and 900–1200 kg/m^3^, respectively. However, no clear trend was obtained by the KDE jointplot between SP content and ${D}_{nssm}$ classifications. As illustrated in Fig. 8, concrete mixtures should have a fresh density range of 2100–2500 kg/m^3^ to resist the chloride attack. Finally, the KDE distribution jointplot revealed that high chloride-resistant concrete samples have a concrete compressive strength of 45–60 MPa.

**Figure 9:**
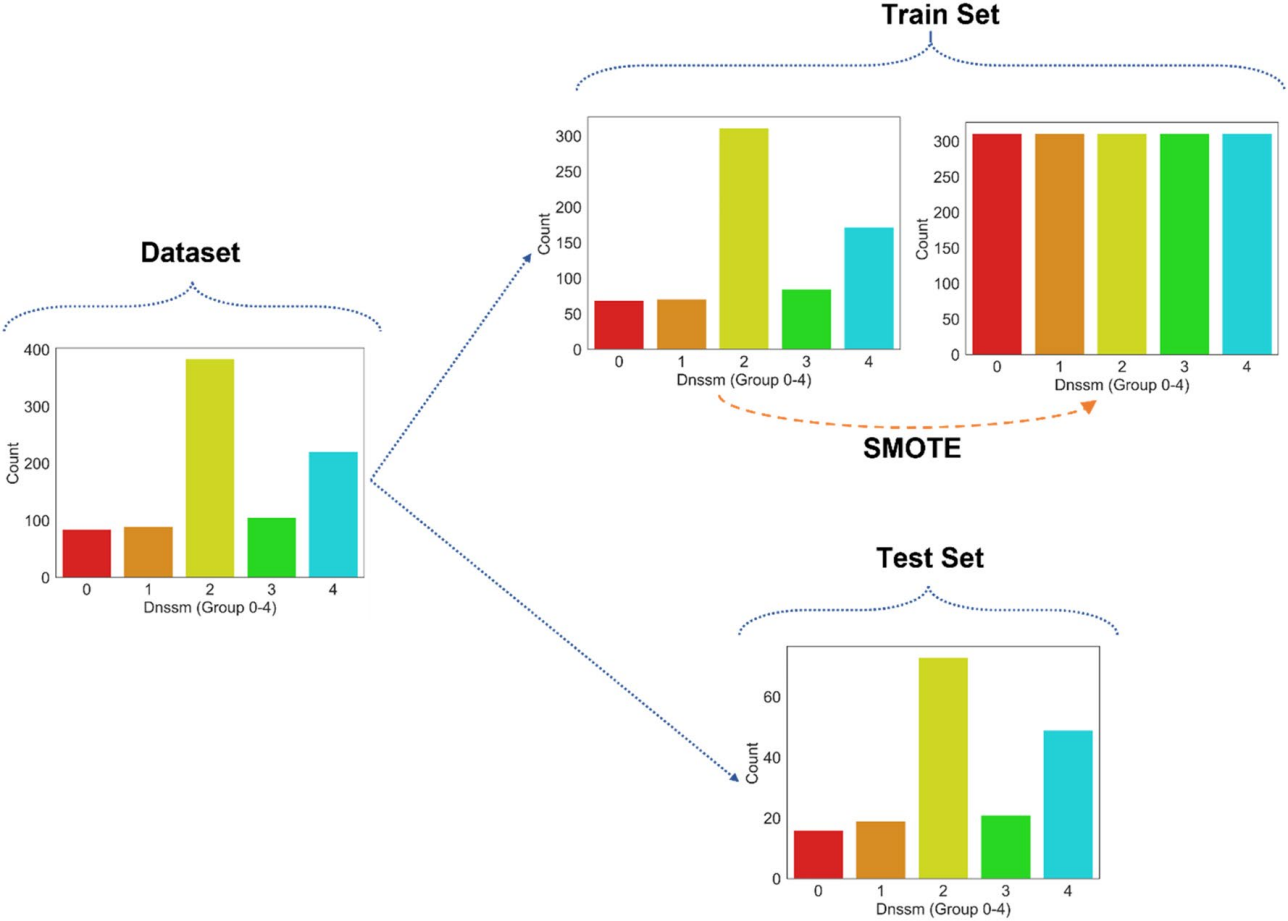
Synthetic Minority Oversampling Technique (SMOTE) on training data.

Data visualization plots revealed that the number of datasets for ${D}_{nssm}$ plays a significant role in the efficiency of the analysis. As shown in Fig. 9, category number 2 has the highest number of datasets ${D}_{nssm}$. Using this dataset to train the machine causes a misleading prediction model. Accordingly, the synthetic minority oversampling technique (SMOTE) is used in the present study to increase the number of datasets for training the machine. This technique is one of the most frequently used oversampling approaches to achieve a balanced training dataset. This technique balances the class distribution by erratically increasing minority class instances by duplicating them. On the other hand, using SMOTE causes the generation of 1750 training datasets by having an equal number of 350 datasets for each ${D}_{nssm}$ classification. Only 80 percent of all datasets were considered for training. It is worth mentioning that SMOTE was only used for training machines, while real experimental datasets were used for testing.

**Figure 10:**
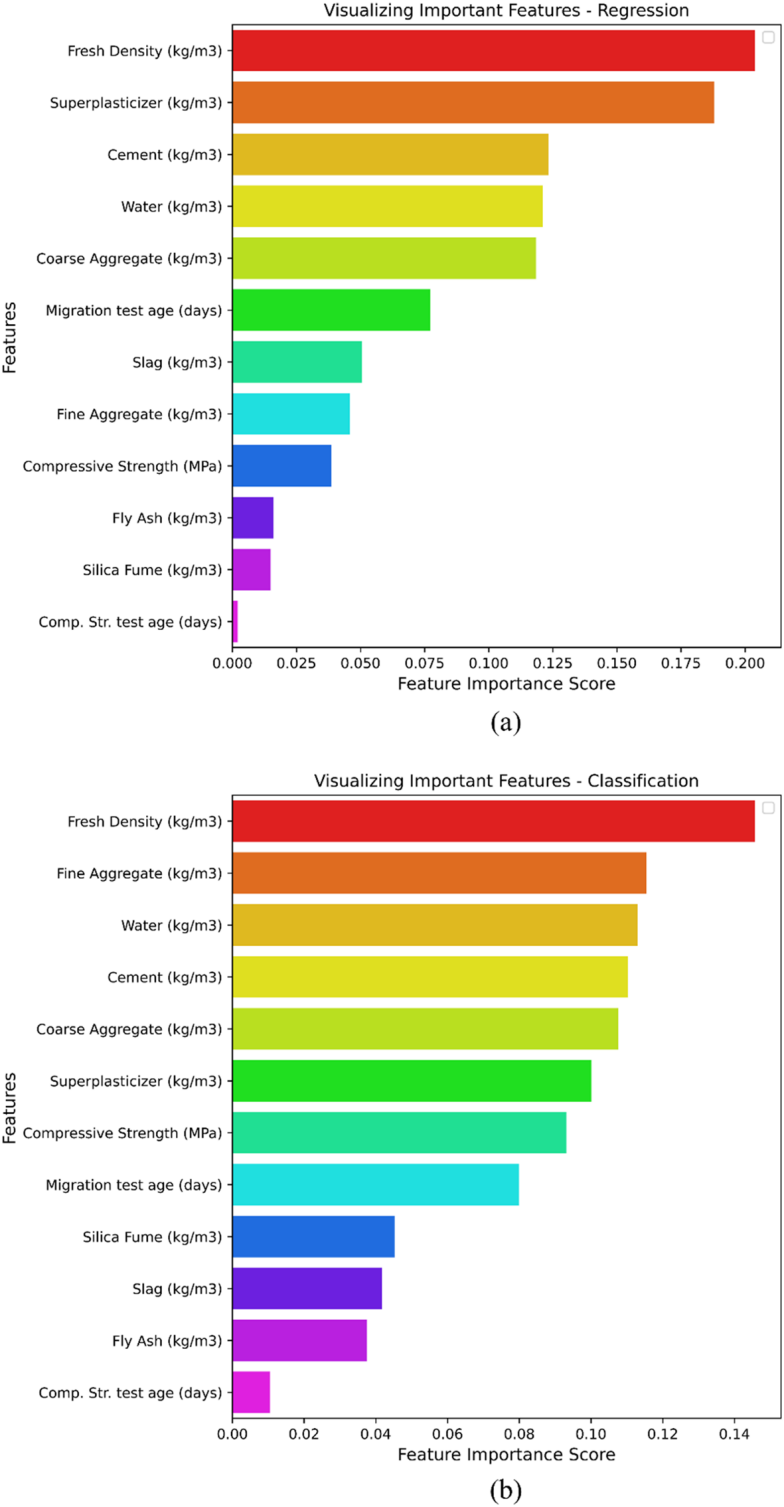
Feature importance techniques for: (**a**) Regression; (**b**) Classification.

Prior to commencing the machine learning model in data visualization, it is imperative to conduct a thorough examination of the feature importance plots for both regression and classification methodologies, as depicted in Fig. 10a,b correspondingly. Feature importance score represents the significance of each input feature for a ${D}_{nssm}$ model. A specific feature with a higher score has a more significant influence on the predicting model of ${D}_{nssm}$. Based on the regression approach, results of feature important show that superplasticizer dosage, fresh density, and water content have the highest impacts on the predicting regression model of ${D}_{nssm}$. However, compressive strength test age and FA have the lowest effects on the regression model of ${D}_{nssm}$ (Fig. 10a). Fresh density, coarse aggregate content, and fine aggregate content are the most crucial variables that affect the predicting model in the classification approach. Similar to the regression approach, FA and compressive strength test age have no effective impact on the predicting model of the classification approach. The importance of fresh density was confirmed in both regression and classification approaches.

### Model learning

**Figure 11:**
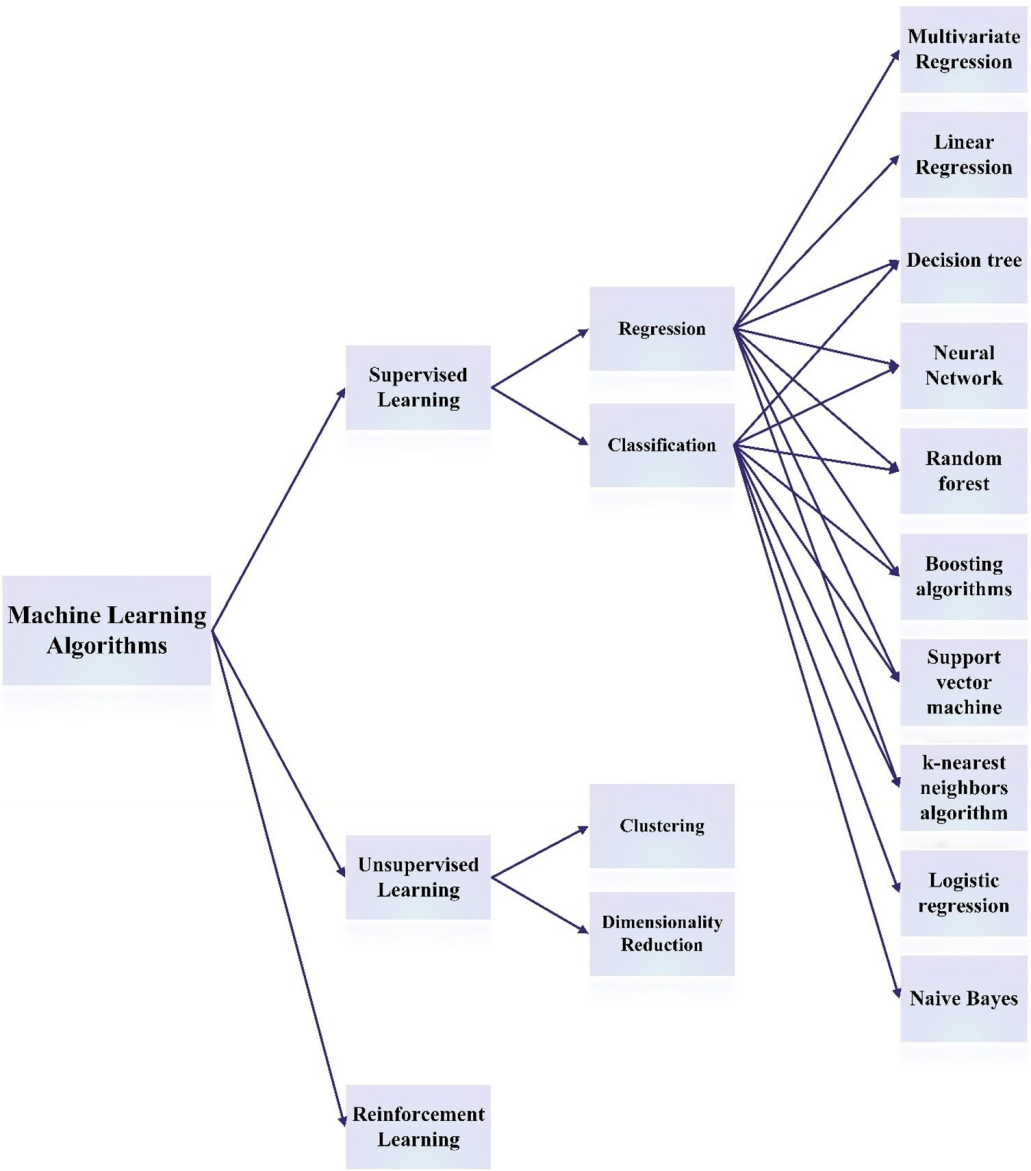
Machine learning algorithms.

**Figure 12:**
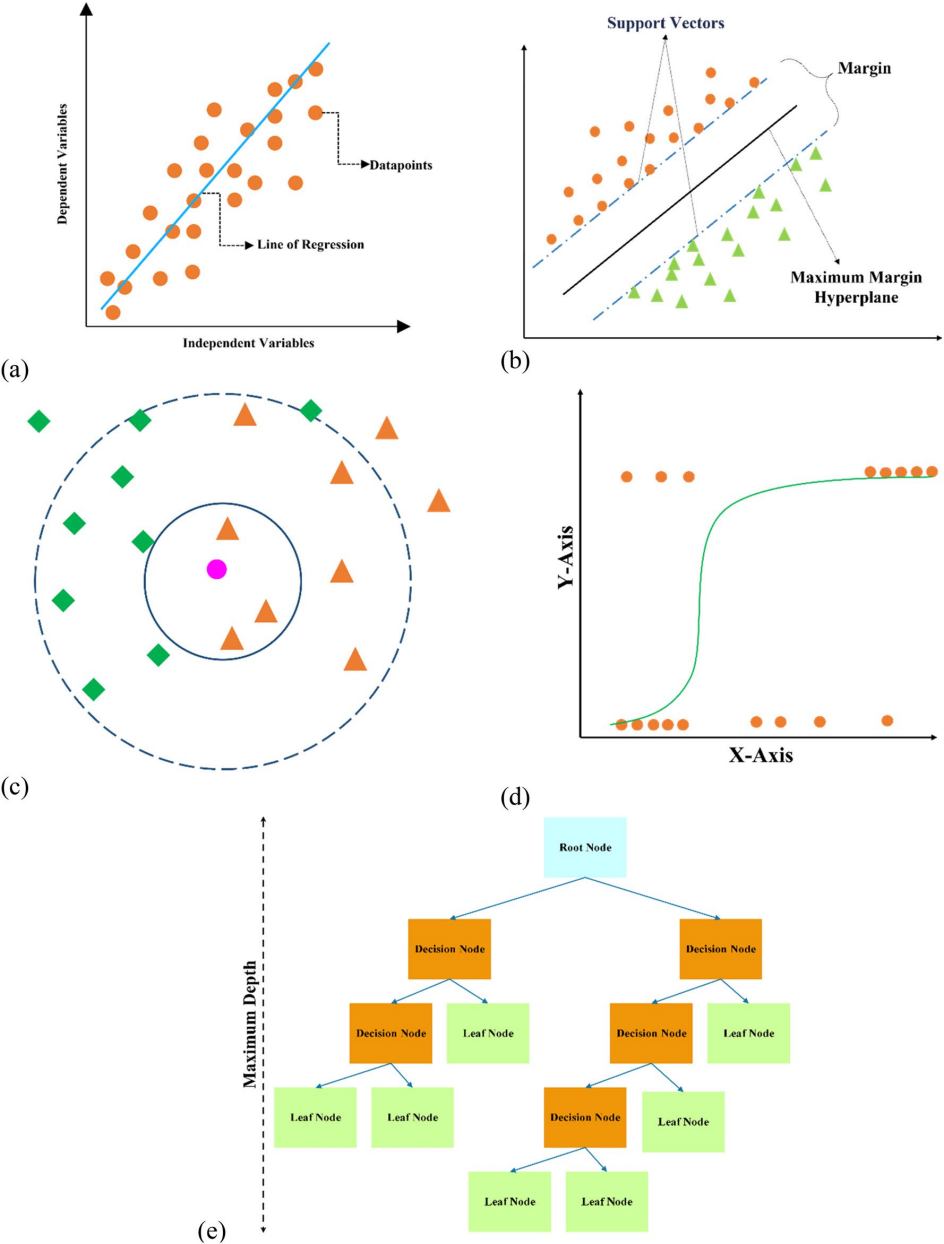
Schematic illustrations of regression and classification models: (**a**) Linear regression; (**b**) Support vector machines (SVM); (**c**) K-nearest neighbors (KNN); (**d**) Logistic regression; (**e**) Decision tree.

As shown in Fig. 11, the ML method contains three types of models, including (1) supervised learning, (2) unsupervised learning, and (3) reinforcement learning. Each type has various algorithms. Supervised learning is used in the present study to introduce a unified predicting model of ${D}_{nssm}$. Supervised techniques adjust the model to reproduce the target variable known from a training dataset. This type of ML has two main models, including (a) Regression and (b) Classification. The regression technique intends to reproduce the output or target value, while classification provides a model to produce the class assignments or data encoding. It can predict the target value, and the data is divided into different categories denoted as “classes.” Elastic Net, Lasso, Linear Regression, Ridge, Random Forest, KNN, Decision Tree, SVR, and XGB are different regression models used in the present study. Decision Tree, KNN, Logistic Regression, XGBoost, LightGBM, Random Forest, and Support Vector Machine are classification models considered in the present study. Schematic illustrations of some regression and classification models are shown in Fig. 12. Generally, linear Regression consists of four different linear regression models, including Simple, Ridge, Lasso, and Elastic Net. Linear Regression aims to predict a dependent output (or target) through different independent variables. Accordingly, this regression technique determines a linear relationship between a response variable and the other predictor variables (Fig. 12a). Support Vector Machines (SVM) as a robust classification and regression model convert the data into a linear decision space to classify datasets, even when the data are not linearly distinguishable. Support vectors are datasets nearer to the line (or hyperplane) and affect the location and direction of the hyperplane. A separator between the categories is created, and then the data are converted to draw the separator as a hyperplane (Fig. 12b). K-Nearest Neighbors (KNN) is a non-parametric and non-linear data classification method using proximity to perform classifications or estimations about the grouping of an individual data point (Fig. 12c). Logistic Regression is essentially a non-linear extension of linear Regression, allowing us to handle classes in a classification problem. This is achieved by categorizing estimates into a given class based on a likelihood threshold (Fig. 12d). Logistic Regression is a statistical analysis method for calculating the probability of a binary outcome, such as yes or no, based on former findings of a dataset. Logistic Regression affords discreet output. Finally, Decision Tree is a hierarchical demonstration of the outcome space where each node signifies a selection or an act. This model is used to classify or estimate how a former set of queries were responded to (Fig. 12e).

### Model evaluation

Different statistical criteria are used in the present study to determine the efficiency of regression and classification models. For the regression models, different coefficients are used in the present study, as follows:

3 $$RMSD = \sqrt {\frac{{\mathop \sum \nolimits_{i = 1}^{N} \left( {x_{i} - \hat{x}_{i} } \right)^{2} }}{N}}$$

4 $$MAE = \frac{{\mathop \sum \nolimits_{i = 1}^{n} \left| {y_{i} - x_{i} } \right|}}{n} = \frac{{\mathop \sum \nolimits_{i = 1}^{n} \left| {e_{i} } \right|}}{n}$$

5 $$R^{2} = 1 - \frac{RSS}{{TSS}}$$

6 $$MSE = \frac{1}{n}\mathop \sum \limits_{i = 1}^{n} \left( {Y_{i} - \hat{Y}_{i} } \right)^{2}$$

**Figure 13:**
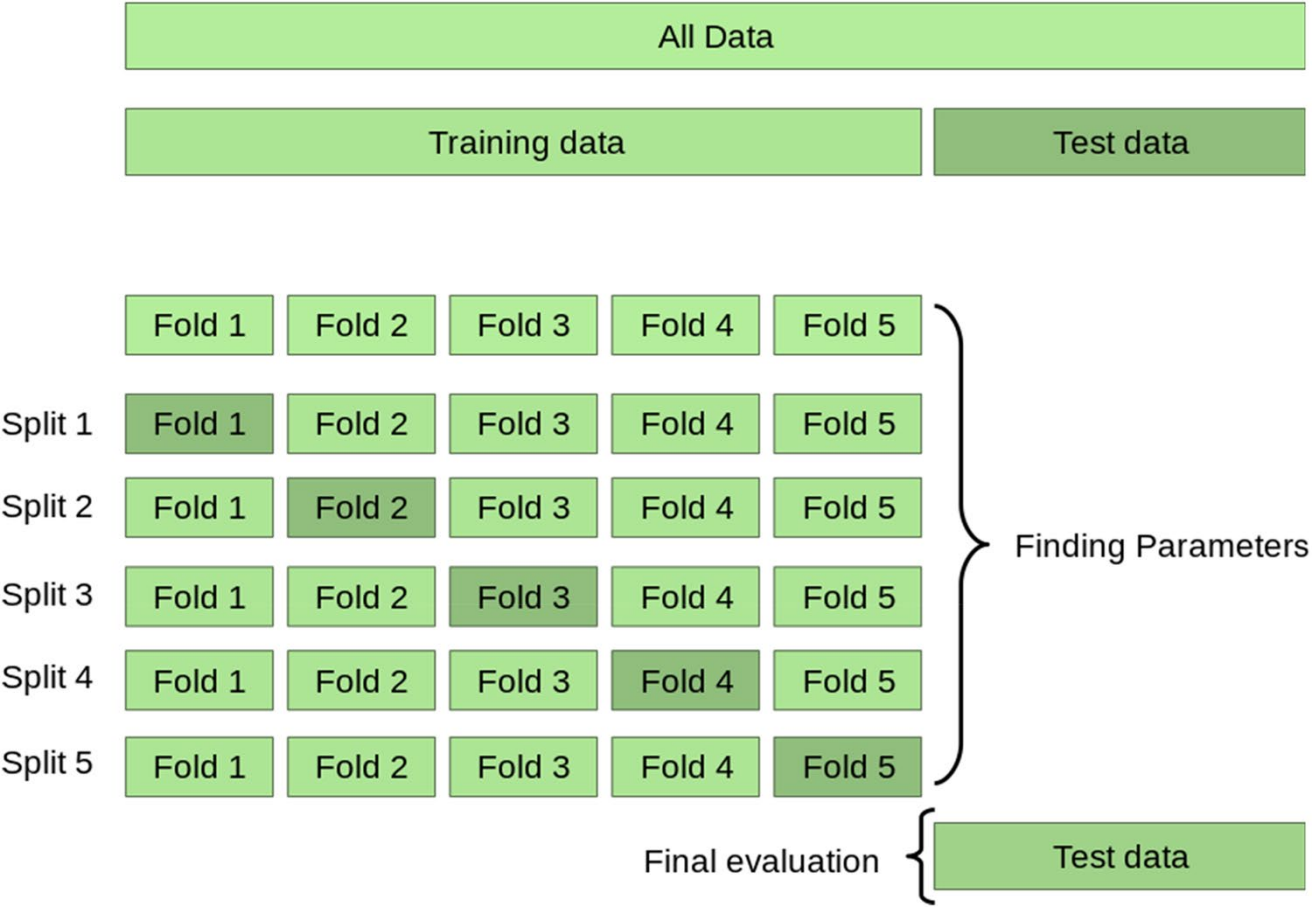
K-fold cross-validation (CV).

**Figure 14:**
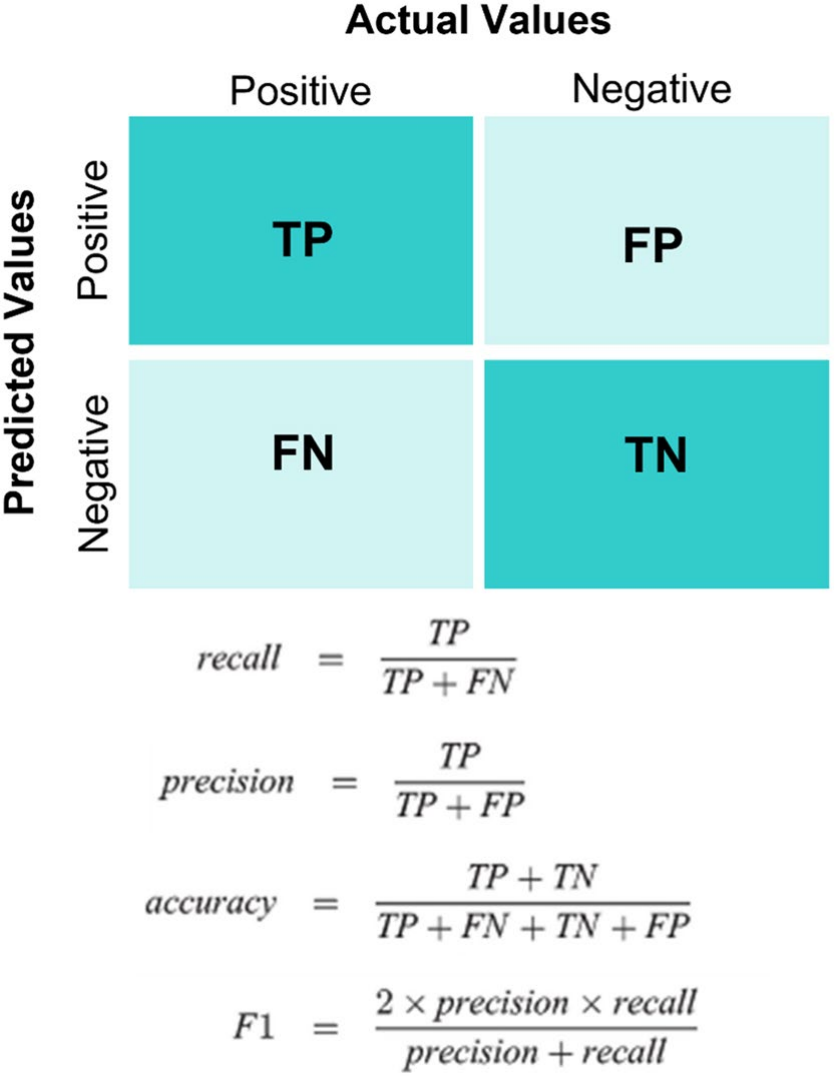
Confusion matrix as a performance measurement for classification models.

Root Mean Square Error (RMSE) is the square root of the error function and should be reduced by the regression models to fit the datasets appropriately. Based on Eq. (3), N is the number of non-missing data points, $${x}_{i}$$ is the actual observations time series, and $${\widehat{x}}_{i}$$ is the estimated time series. As mentioned in Eq. (4), Mean Absolute Error (MAE) is used as the sum of the error values. However, as the absolute value is used instead of squaring it, it is more tolerant of large estimate errors. R Square ($${R}^{2})$$ calculates how much the regression model can describe changeability in a dependent variable. Although It is a respectable measurement to check the fit of dependent variables, no overfitting is considered in $${R}^{2}$$. Based on Eq. (5), RSS and TSS are the sum of squares of residuals and the total sum of squares, respectively. As stated in Eq. (6), MSE is Mean Squared Error, which determines how close is a fitted line to datasets. In this equation, $$n$$ is the number of data points, $${Y}_{i}$$ is observed values, and $${\widehat{Y}}_{i}$$ is the predicted value. Along with these criteria, the accuracy of the regression models is also improved by the K-fold cross-validation (CV) and hyper-parameter tuning techniques (Fig. 13). Generally, the K-fold CV process is used to assess the efficiency of ML models when making estimates on datasets not used during training. CV is a resampling method used to assess ML models on limited data points. This method has a parameter called k that denotes the number of groups into which a given data sample should be divided. The procedure for using this method is mentioned in the flowchart shown in Fig. 2. Regarding the classification models as performance measurement (Fig. 14). Different performance metrics are used based on the confusion matrix to determine the efficiency of the classification models, including precision, recall, F_1_, accuracy, and specificity.

## Results and discussion

**Figure 15:**
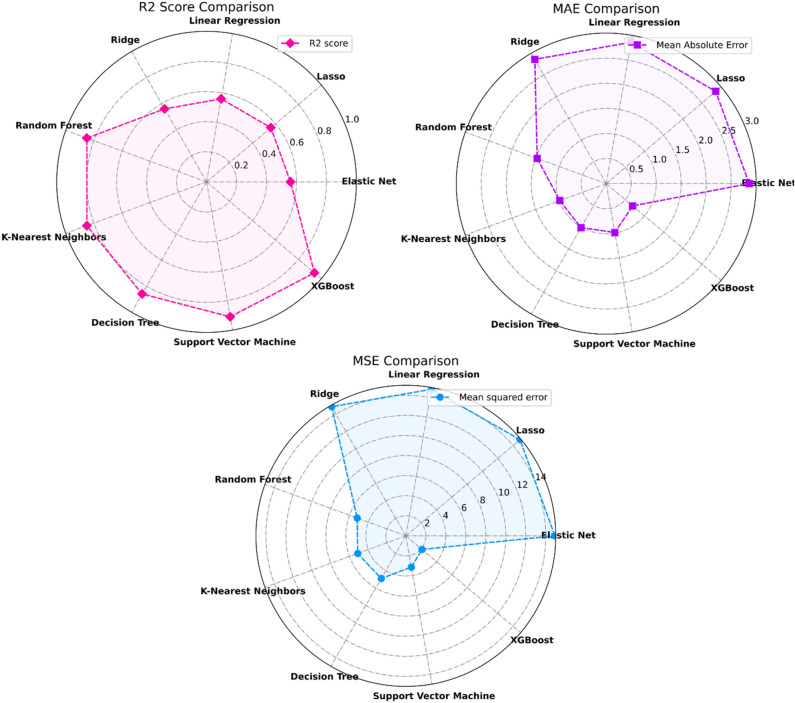
Performance of regression models in ML method for predicting ${D}_{nssm}$.

**Figure 16:**
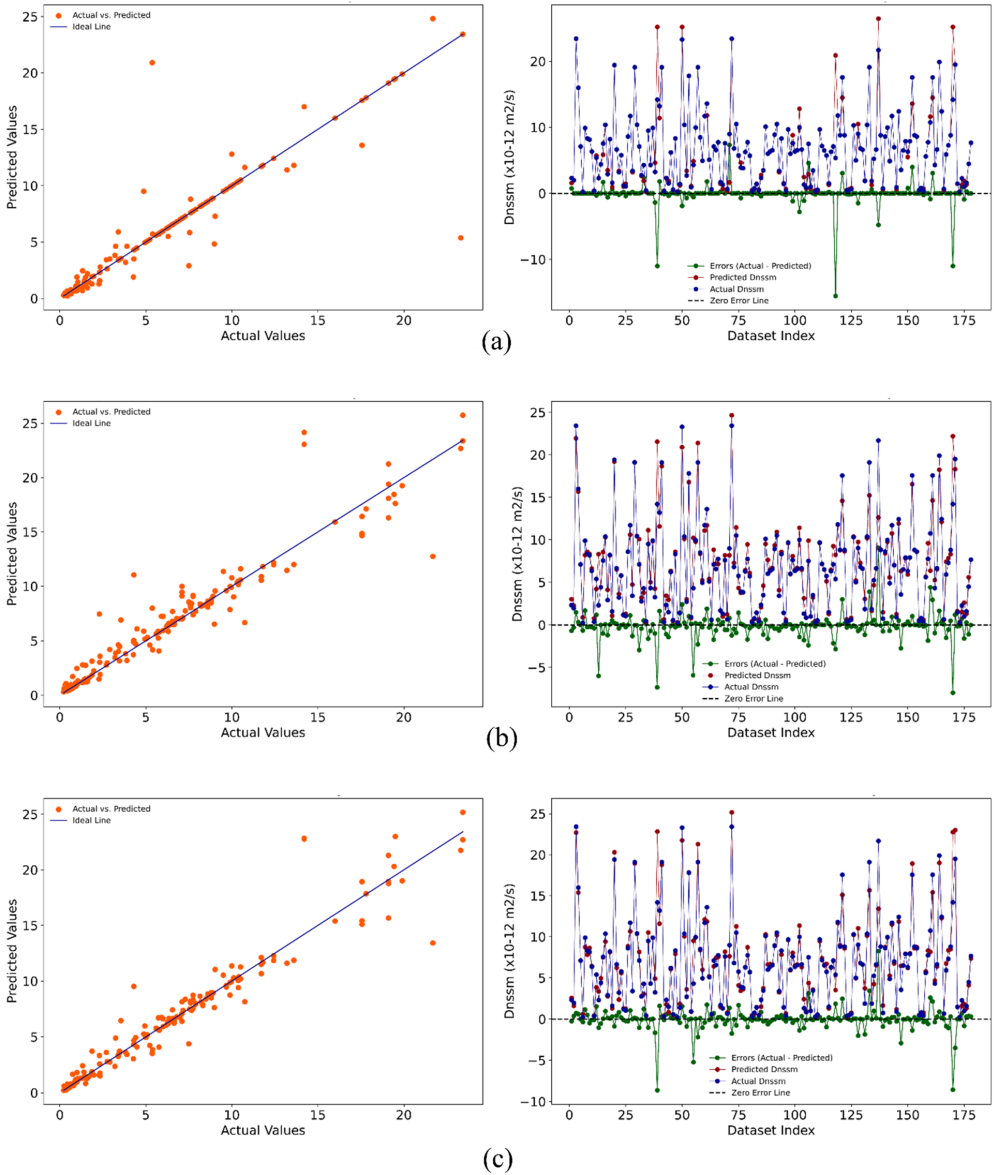
The relationship between the actual and predicted results and error graph: (**a**) decision tree; (**b**) Random Forest; (**c**); XGBoost.

The performance of the regression model in the ML method is summarized in Fig. 15. Figure 16 displays both the error graph and the predicted versus actual graph. The evaluation of the relationship between the actual and predicted results, as well as the error graph, was conducted for three algorithms with higher accuracy: Decision Tree, Random Forest, and XGBoost. The results are depicted in Fig. 16a,b,c respectively. Results indicate that among regression models, XGBoost (${R}^{2}=$ 0.94) and SVR (${R}^{2}=$ 0.94) show the highest accuracy. Also, ML analysis revealed that Elastic Net, Lasso, Linear regression, and Ridge could not precisely predict the ${D}_{nssm}$. Moreover, accepted ${R}^{2}$ score was found for Random Forest, KNN, and decision tree. It is worth mentioning that achieving a reliable model (XGBoost) with this high ${R}^{2}$ score for this content of datasets (1037 numbers) showing the efficiency of the proposed unified method followed by the present study.

**Figure 17:**
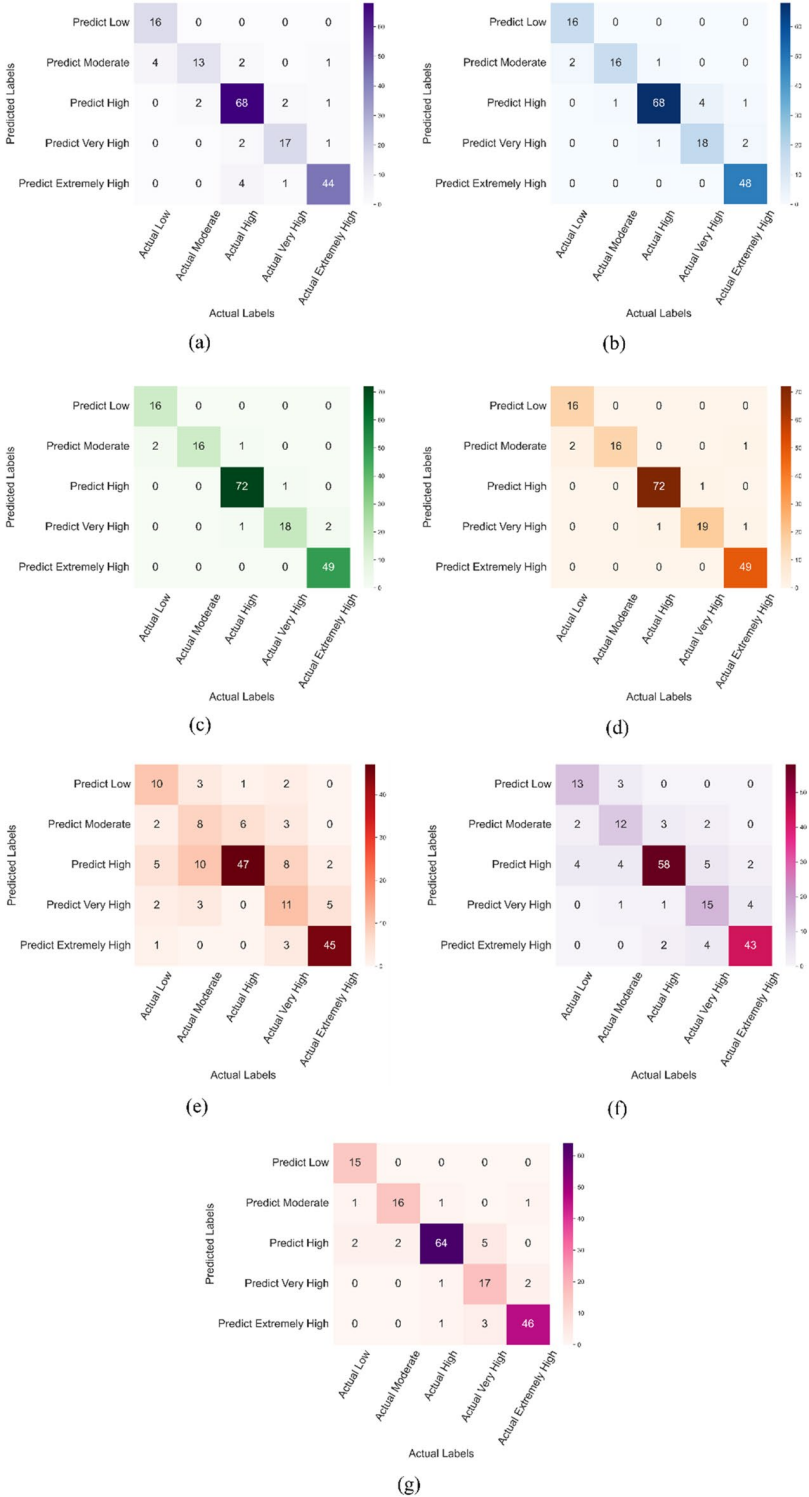
Performance of classification models using confusion matrix: (**a**) Support vector machine (SVM); (**b**) random forest; (**c**) LightGBM; (**d**) XGBoost; (**e**) Logistic regression; (**f**) KNN; (**g**) Decision tree.

The performance of the classification models by the confusion matrix is shown in Fig. 17. Regarding the SVM model, results show an accuracy of 0.89 with the highest efficiency for Class 0 (based on the classification of Table 1). As shown in Fig. 17a, the first row shows all datasets of the first category, where all of them (16 datasets) were successfully predicted, without any sample out of accuracy. For Category 3 (Predict High), the SVM model appropriately predicted 68 datasets (from 73 numbers), showing high accuracy. The average accuracy of 0.93 was found for the Random Forest model with the highest performance for the categories Low & Extremely High with accuracy factors of 1.0 and 0.96, respectively (Fig. 17b). The LightGBM model shows an accuracy of 0.96 (Fig. 17c). Almost all categories for this model have F_1_ scores higher than 0.90. The XGBoost model also has a high accuracy of 0.97, the maximum accuracy among other classification models (Fig. 17d). The lowest accuracy was found for the Logistic Regression model with an accuracy of 0.68, where none of the categories reached 0.90 Accuracy (Fig. 17e). Results also showed that the accuracy of the KNN model (0.79) is lower than that of the Decision-Tree model (0.88), as shown in Fig. 17f,g.

**Figure 18:**
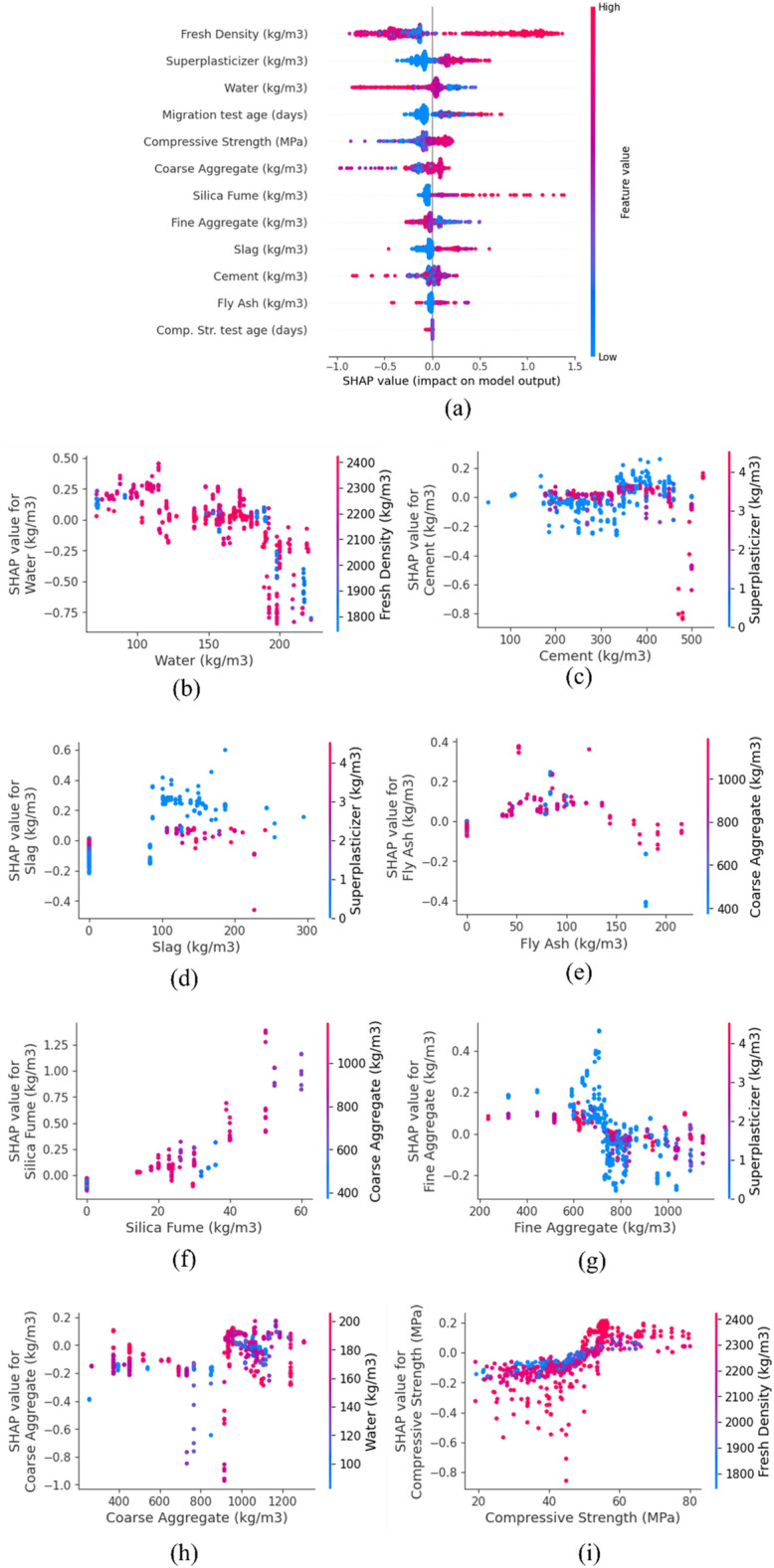
Results of SHapley Additive exPlanations (SHAP) approach.

After checking the performance of each predicting model, the SHAP (SHapley Additive exPlanations) approach is used in the present study to determine the sensitivity of the predicting model considering different variables. SHAP is a game hypothetical and mathematical method to clarify the ML model's output by measuring each feature's influence on the prediction. As shown in Fig. 18a, the SHAP value indicates that high content of fresh density has a considerably positive impact on the predicting model, while high water contents have an adverse influence. Moreover, a high dosage of SP increases the possible effect on the predicted model. SHAP results also revealed that high contents of SF and migration test age positively impact the predicting model. Based on SHAP analysis, lower concrete compressive strength has a negative effect on the predicted ${D}_{nssm}$. Furthermore, it can be deduced from the SHAP value that the lower content of fine aggregate positively affects the predicted model, while the lower content of coarse aggregate negatively influences the model. The high content of cement and slag was also found to adversely affect the ${D}_{nssm}$ model. However, another trend was found for FA so that high content causes a positive influence on the model. No trend was found for the compressive strength test age. Another valuable finding of the SHAP approach is finding the most critical relationship between the features. For instance, as shown in Fig. 18b, fresh density affects the influence of water on the SHAP value. Also, SP content controls the effect of cement on the SHAP value, so that for cement lower than 350 kg/m^3^, the high content of the SP has a higher impact on the predicting model, while this trend is vice versa for a higher content of cement (Fig. 18c). SHAP analysis found that SP dosage affects the interaction between slag content and the predicting model (Fig. 18d). As shown in Fig. 18e, higher coarse aggregate content results in a better relationship between FA and predicted ${D}_{nssm}$. In this field, SHAP analysis showed that coarse aggregate affects the SF influence on the predicting model so lower coarse aggregate content causes a more proper relation between SF and the predicting model (Fig. 18f). In this context, Fig. 18g demonstrates that for fine aggregate content lower than 800 kg/m^3^, low SP dosage causes a more positive impact of fine aggregate on the model, while this trend changes for fine aggregate higher than 800 kg/m^3^. SHAP analysis depicts that water content controls the effect of coarse aggregate on the predicting model (Fig. 18h), so that high content of water causes a higher impact of coarse aggregate on the ML model for coarse aggregate content lower than 800 kg/m^3^. Finally, SHAP analysis showed that fresh density considerably affects the compressive strength-predicting model relationship, so for compressive strength lower than 50 MPa, high fresh density results in a more devastating effect on the predicting model. However, this trend changes for ${f}_{c}\ge$ 50 MPa where high fresh density improved the effect of compressive strength on the predicting model (Fig. 18i).

## Validation process

The present study incorporated an additional validation process to assess the efficacy of the current research. The outcomes of this validation procedure are concisely presented in Table 6. In the current study, a total of 886 datasets were utilized, following the data cleaning process. These datasets were employed for the regression algorithm, as depicted in Fig. 15, and the classification algorithm, as illustrated in Fig. 17. The model outputs were found to be associated with all of the datasets.

**Table 6:** Results of the validation process.

Water (kg/m^3^)	Cement (kg/m^3^)	Slag (kg/m^3^)	Fly ash (kg/m^3^)	Silica fume (kg/m^3^)	Fine aggregate (kg/m^3^)	Coarse aggregate (kg/m^3^)	Super plasticizer (kg/m^3^)	Fresh density (kg/m^3^)	f_c_ (MPa)	Dnssm (× 10^−12^ m^2^/s)					
										Actual value		Predicted value		Error (%)	
										Regression	Classification	Regression	Classification	Regression	Classification
96.11	222.48	148.32	0	0	730.32	1096.37	0	2301.85	45.81	5.09	2	4.66	2	-9	0
120.46	250.96	83.65	0	0	768.89	1105.87	0	2332.61	34.82	17.56	0	18.02	0	3	0
153.07	355.97	0	0	0	637.18	1187.15	0	2280.55	42.58	9.2	2	10.01	2	8	0
95.79	231.38	124.59	0	0	725.58	1117.73	0	2300.25	49.3	7.16	2	7.02	2	− 2	0
114.86	261.04	101.45	0	0	745.16	1013.91	0	2244.83	37.34	9.33	2	8.9	2	− 5	0
97.45	226.63	151.29	0	0	759.99	1045.35	0	2292.24	44.29	7.12	2	7.13	2	0	0
99.78	227.82	151.88	0	0	671	1061.37	0	2247.39	45.09	6.98	2	6.98	2	0	0
153.24	373.76	0	0	29.66	644.3	1072.05	0	2258.6	47.68	12.42	1	12.43	1	0	0
82.23	195.78	160.18	0	0	702.52	1116.74	0	2292.24	46.57	6.23	2	6.22	2	0	0
115.54	281.81	0	74.16	23.73	731.51	1017.47	0	2240.98	39.64	16	0	15.92	0	0	0
115.54	281.81	0	74.16	23.73	742.78	1020.44	0	2240.98	39.64	13.59	1	13.73	1	1	0
95.63	255.7	0	45.68	0	591.5	767.11	0	2338.7	46.24	9.02	2	8.89	2	− 2	0

After conducting an analysis of feature importance and data visualization, as well as evaluating various models, a new dataset consisting of a 28-day test age was chosen as the input dataset for the website. This test age was selected due to its prevalence among civil engineers. In order to validate and confirm that certain data points were not observed by the algorithm, a subset of 12 numbers from the datasets were completely isolated from the dataset utilized for the final validation procedure.

As indicated in Table 6, the regression model yielded a deviation below 9%. Furthermore, the classification model exhibits a high level of precision in its predictions, displaying minimal deviation. As a result, the proposed machine learning classification model successfully forecasts precise categories.

**Figure 19:**
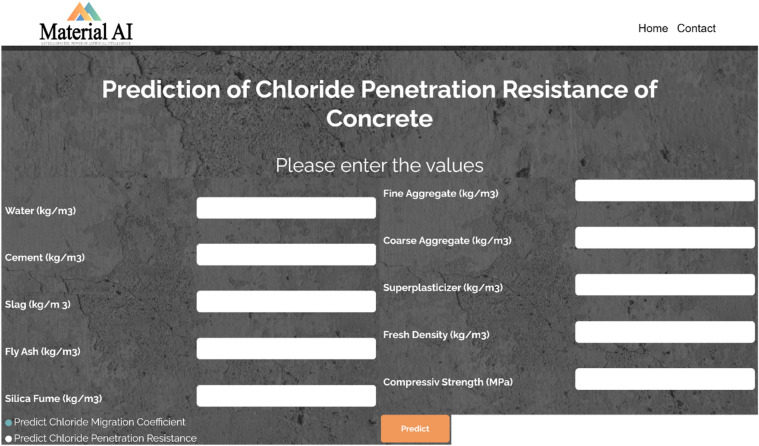
Free access ML predictive model for concrete durability checking designed by the present study (Link of this model https://materialai.ir/).

The present study has developed a free-access machine learning predictive model for concrete durability checking, as depicted in Fig. 19. This model can be accessed through the productive link "http://materialai.ir". Researchers and engineers involved in the development of various concrete mixtures have the ability to verify the durability of their mixtures through online platforms. The utilization of extensive datasets and the inclusion of multiple validation stages in the current study make this resource applicable to a wide range of concrete types.

## Concluding remarks

To present a reliable and efficient prediction model for ${D}_{nssm}$, an extensive experimental database containing 1037 datasets was gathered in the present study containing different types of concrete mixtures. As existing predicting models for chloride resistance have two main problems, the limited dataset considered and missing input variables causing a reduction in their efficiency, a unified data-cleaning technique was used to improve the consistency of the datasets. Different ML methods of Regression and classification were used. Simple linear Regression, Ridge, Lasso, Elastic Net, Support Vector Machine (SVM), Random Forest, LightGBM, XGBoost, Logistic Regression, KNN, and Decision-Tree are the models used to predict the ${D}_{nssm}$. Water, cement, slag, fly ash, silica fume, fine aggregate, coarse aggregate, superplasticizer, fresh density, compressive strength, compressive strength test age, and migration test age are the variables considered as independent predictors. Also, the SHAP approach was used to check the importance of each variable in the predicting model. Generally, results showed that XGBoost and SVR (as regression models) could appropriately predict the ${D}_{nssm}$ with ${R}^{2}$ score higher than 0.90. Moreover, among the classification models, LightGBM and XGBoost showed the highest accuracy factor. SHAP analysis also indicates that there is a controlling effect between impacts of cement–water, slag-SP, FA-coarse aggregate, SF-fine aggregate, compressive strength-fine aggregate, fresh density-coarse aggregate, and fresh density-compressive strength on the predicting ${D}_{nssm}$ model.

### Supplementary Information

Supplementary Information.

## Data Availability

The authors confirm that the data supporting the findings of this study are available within the article and its Supplementary material. Raw data supporting this study's findings are available from the corresponding author upon reasonable request.

## Abbreviations

AEA: Air-entraining agent
AI: Artificial intelligence
ANN: Artificial neural network
BP: Backpropagation
Cs: Surface chloride concentration
CART: Classification and regression trees
CNI: Calcium nitrite-based corrosion inhibitor
D_nssm_: Non-steady-state migration coefficients
DCT: Dataset-cleaning technique
EDT: Ensemble decision tree
EnKF: Ensemble kalman filter
FA: Fly ash
${f}_{c}$: Concrete compressive strength
GEP: Gene expression programming
GGBFS: Ground granulated blast furnace slag
GP: Genetic programming
HCFA: Concrete containing high calcium fly ash
HPC: High-performance concrete
HRWR: High range water reducer
HSC: High strength concretes
MARS: Multivariate Adaptive Regression Splines
MGGP: Multi-Gene Genetic Programming
MK: Metakaolin
ML: Machine learning
MPMR: Minimax probability machine regression
NC: Normal concrete
PEC: Passed electric charge
PSO: Particle swarm algorithm
RA: Recycled aggregate
RAC: Recycled aggregate concrete
RC: Reinforced concrete
RCPT: Rapid chloride penetration test
RF: Random forest
SCC: Self-consolidating concrete
SCMs: Supplemental cementitious materials
SF: Silica fume
SHAP: SHapley Additive exPlanations
SP: Superplasticizer
t: Time
VMA: Viscosity modifying admixture
W/B: Water-to-binder ratio
W/C: Water-to-cement ratio
X: Position from the concrete surface
