# Supplementary material for: An efficient machine learning approach for predicting concrete chloride resistance using a comprehensive dataset
Source: Sci Rep. 2023 Sep 12;13:15024. doi: 10.1038/s41598-023-42270-3 (PMC10497559; doi:10.1038/s41598-023-42270-3)
Supplement: Supplementary file 1 — Supplementary Information. [file 41598_2023_42270_MOESM1_ESM.docx]

**Supplementary file of**

**“An efficient machine learning approach for predicting concrete chloride resistance using a comprehensive dataset”**

By

Maedeh Hosseinzadeh^1^, Seyed Sina Mousavi^2^, Alireza Hosseinzadeh^3^ and Mehdi Dehestani^4^*

| 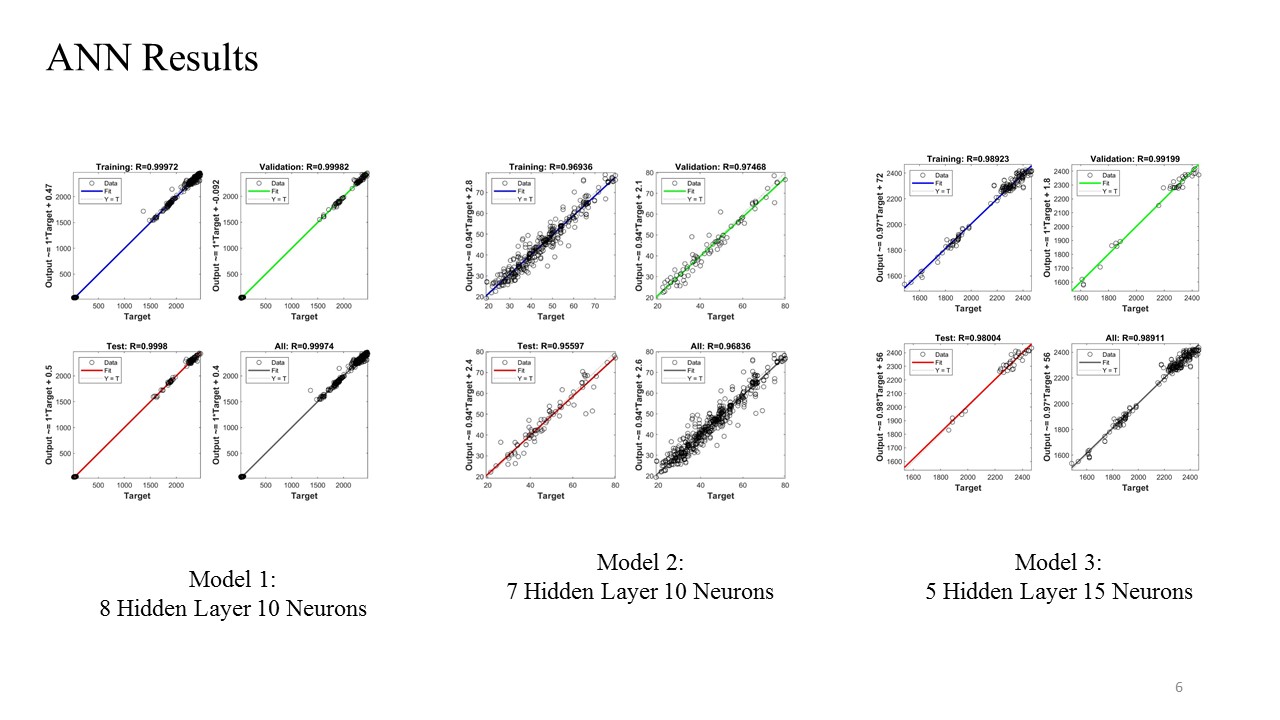 |
| --- |
| **Fig. S1** ANN Results for the dataset-cleaning technique (DCT) |
